# Cross-species validation of a human age-related hearing loss candidate *KLHDC7B* as essential for mammalian hearing

**DOI:** 10.1038/s42003-025-09349-1

**Published:** 2025-12-17

**Authors:** Alexandra M. Kaufman, Benjamin Silver, Roberto A. Donnianni, Carlos Aguilar, Lingzi Niu, Daniel Johnson, Anwen Bullen, Alma Corona, Benjamin J. van Soldt, Nilay Vora, Gervasio Batista, Luz Cortes-Burgos, Jacqueline Copeland, Elika Fallah, Norman Zhang, Marina Lehmkuhl, Sarah Cancelarich, Kara Campos, Daniela Di Battista Miani, Jaylen Mumphrey, Susan D. Croll, Johnathon R. Walls, Mary Germino, Michael R. Bowl, Meghan C. Drummond, Sally J. Dawson

**Affiliations:** 1 Regeneron Pharmaceuticals Inc, Tarrytown, NY USA; 2 UCL Ear Institute, University College London, London, UK; 3 Medical Research Council Harwell Institute (Mammalian Genetics Unit and Mary Lyon Centre), Harwell, Oxfordshire UK

**Keywords:** Medical genetics, Neurodegenerative diseases, Hair cell

## Abstract

Although age-related hearing loss (ARHL) is the most common sensory loss in older adults, underlying mechanisms remain unclear. Recent genome-wide association studies (GWAS) linked variation in several genes with ARHL risk, including *KLHDC7B*, a gene of unknown function not previously linked to hearing. We demonstrate *Klhdc7b* is expressed exclusively in sensory hair cells in mouse cochlea. Utilizing two independent mouse knockout models (*Klhdc7b*^*IMPC-/-*^ and *Klhdc7b*^*Regn*Δ/Δ^) we find that absence of *Klhdc7b* leads to severe early-onset, progressive hearing loss. Hair cells appear to develop normally, but outer hair cells are progressively lost from base-to-apex of the cochlea, a common pathology in ARHL. These results suggest KLHDC7B is required for maintenance rather than development, of auditory function. The validation in mouse of a human ARHL GWAS association suggests other novel candidates should be investigated. Our work provides two mouse models to study KLHDC7B function, and for development of therapeutic tools for ARHL.

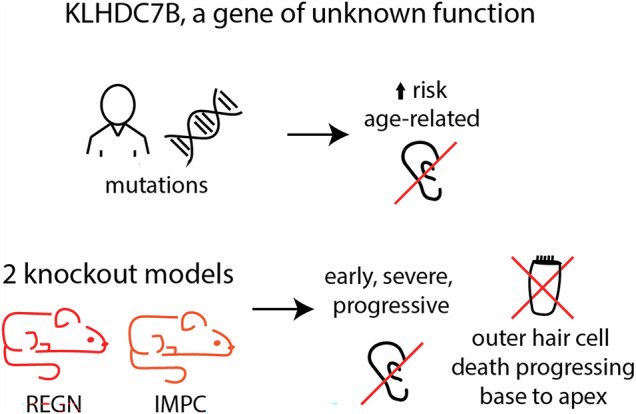

## Abstract (web summary)

Using two independent knockout mouse models, the authors show that loss of *Klhdc7b*, a candidate for susceptibility to age-related hearing loss in humans, results in an early and progressive hearing loss, confirming *Klhdc7b*’s role in the maintenance of hearing.

## Introduction

Age-related hearing loss (ARHL) is a common condition that can lead to social isolation, depression, and a decrease in the quality of life. Furthermore, hearing loss in mid-life has recently been found to be associated with an increased risk of subsequent dementia diagnosis. In 2024, the Lancet Commission published an update on their previous reports in 2017 and 2020 which again pinpointed hearing loss in mid-life as the largest potential modifiable risk factor for dementia^1–3^. The number of older people with hearing loss and dementia is rising as the aging population increases^4^. Age-related hearing loss is the most common sensory loss with more than 50% of people aged 60–69, and over 80% of those aged 85 years and older having some hearing loss that affects daily communication and would benefit from the use of a hearing aid^5^. It is a complex condition involving a combination of genetic risk factors and exposure to environmental components such as noise exposure and ototoxic drugs. Evidence from early GWAS into ARHL suggested that genetic effects were likely to be small or due to rare alleles, and these studies largely did not identify genome-wide significant associations^6–13^. However, several recent GWAS utilizing self-report hearing data in the large UK Biobank Cohort dataset, together with subsequent meta-analysis and replication studies have for the first time identified over 50 genetic loci significantly associated with ARHL risk^14–18^. Furthermore, whole exome sequencing and genome sequencing have identified further candidate genes using rare variant as well as common variant approaches^19^. Some of these genes have known auditory function and/or have been previously linked to congenital deafness, but the majority are novel associations with hearing thereby presenting an opportunity to reveal new pathogenic mechanisms for ARHL.

The strongest association (p = 1.90E-22) with self-reported hearing in the UK Biobank GWAS^16^ is with a valine to methionine (Val504Met) missense variant (rs36062310) within the kelch-domain containing 7b (*KLHDC7B*) gene. A recent meta-analysis of five cohorts identified the same variant in association with hearing loss in adults (p = 4.24E-26)^18^. The same study also utilized whole exome sequencing data to perform a rare variant analysis (minor allele frequency < 0.01), identifying heterozygous predicted loss-of-function variants in *KLHDC7B* associated with increased risk of hearing loss, with an odds ratio of 2.145. Taken together these genetic associations provide a strong rationale for *KLHDC7B* being a novel candidate gene for ARHL, but KLHDC7B has not yet been studied in the auditory system.

Other than its membership in the Kelch-domain containing protein superfamily (due to the presence of highly conserved Kelch domains), very little is known about this protein. Kelch domains are a set of repeating beta-sheet forming subunits that come together to form a tertiary structure known as a beta propeller. Kelch domain containing proteins have diverse subcellular locations and functions. For example, as reviewed in ref. ^20^, in Limulus spermatozoa, α-scruin colocalizes with actin filaments in the acrosomal process, whereas β-scruin, which shares 67% sequence identity with α-scruin, is located within the acrosomal vesicle, which does not contain actin^20,21^. There are only a few publications specifically examining KLHDC7B. It has been shown to be upregulated, yet also hypermethylated, in breast cancer cells^22^, but its biological function remains uncharacterized.

To investigate the role of *KLHDC7B* in hearing, we determined the cell-type specific expression of *Klhdc7b* in the mouse cochlea using qPCR, RNAscope and immunofluorescence finding that within the cochlea, *Klhdc7b* is exclusively detected in hair cells, including inner, outer, and vestibular hair cells. To determine whether KLHDC7B is required for auditory function we utilized two independent knockout mouse models, *Klhdc7b*^*IMPC-/-*^ and *Klhdc7b*^*Regn*Δ/Δ^. The first was generated and maintained on a C57BL/6 N background and the second was maintained on the B6.CAST-*Cdh23*^*753A>G*^ background, a line that was bred with the CAST/EiJ line and then backcrossed to C57BL/6 J to produce a C57BL/6 J line carrying the wildtype allele of *Cdh23* rather than the known age related hearing loss allele in *Cdh23*^23^.

We performed auditory phenotyping by auditory brainstem response (ABR) of wildtype, heterozygous, and homozygote mutant mice in both models at several ages. Mice homozygous for either knockout allele display a similar pattern of severe early hearing loss with some residual hearing progressing to profound deafness at 8-12 weeks of age. Distortion product otoacoustic emission (DPOAE) measurements indicate a similar progression of loss of outer hair cell function in *Klhdc7b*^*Regn*Δ/Δ^ and *Klhdc7b*^*IMPC-/-*^ mice. Histological assessment of cochlear whole mount preparations, scanning electron microscopy (SEM) and transmission electron microscopy (TEM) from these mice indicate that outer hair cells develop normally, but begin to die at around the same time as onset of hearing, around two weeks after birth. Taken together, our results confirm KLHDC7B is required for auditory function and suggest a role in outer hair cell maintenance beginning at the onset of hearing in mice, a role that is consistent with its implication as an ARHL susceptibility gene in humans.

## Results

### *Klhdc7b* is expressed in the inner ear and other mouse tissues

**Fig. 1:**
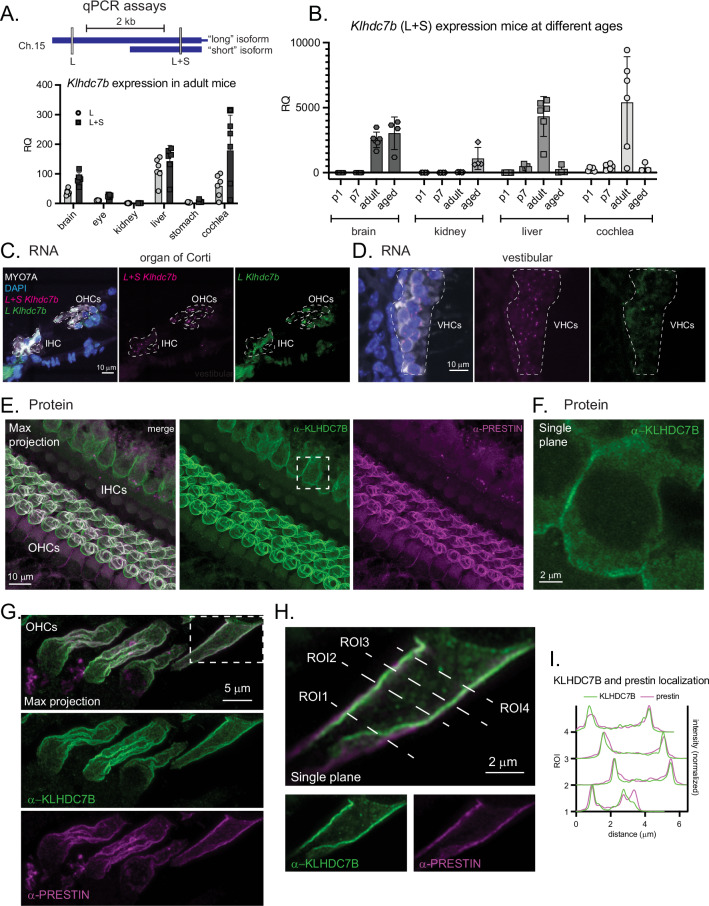
*Klhdc7b* is expressed in cochlear hair cells. **A** qPCR primer/probe sets were designed against the mouse long isoform *(L)* and overlapping portions of the long and short (*L* + *S*) isoforms (schematic, top). Bottom: qPCR using *Klhdc7b L* and *L* + *S* probes in cDNA derived from various adult mice tissue. RQ for each sample is normalized to *Drosha*, a housekeeping gene, and shown compared to mean of adult kidney as a calibrator. **B** qPCR of L + S isoform expression of cDNA derived from mouse tissues at different ages. RQ is normalized to *Drosha*, a housekeeping gene, then compared to mean of P1 kidney. P1, postnatal day 1. P7, postnatal day 7. P1 and P7. For **A**, **B** statistical comparisons are presented in the Supplementary Data 1 statistics file due to the high number of comparisons. Adult tissue was collected from 6 animals, ages 5 females aged 11–14 weeks and 1 male age 24 weeks. Aged mice were 4 animals ages 63–73 weeks. For P1 and P7, n = 5 mice, unsexed. **C**, **D** 40x magnification confocal images of RNA scope probes recognizing the overlapping (L + S *Klhdc7b*, magenta) and long (L *Klhdc7b*, green) portions of the transcripts. MYO7A immunofluorescence (hair cell marker, gray) and DAPI (nuclei, blue) in adult cochlea (female, age 13 weeks). White dashed lines show hair cell outlines. Left, merge; middle, L + S *Klhdc7b*; right, L *Klhdc7b*. **C** organ of Corti. **D** vestibular hair cells. **E** 63x Airyscan maximum intensity projections of a whole mount mouse organ of Corti stained with KLHDC7B (green) and prestin (magenta). **F** Single plane of IHC boxed in (**E**). 6 week old heterozygous (*Klhdc7b*^*Regn*+/Δ^) male mouse. See Fig. 2 for mouse model details. **G** 63x Airyscan maximum intensity projections of outer hair cells showing immunofluorescence against KLHDC7B (green) and prestin (magenta) with individual channels below. **H** A single plane of the OHC boxed in (**G**) with individual channels below. Regions of interest (ROIs) were drawn transverse to the hair cell. **I** Intensity relative to the maximum measured in each ROI demonstrates overlapping expression pattern. IHC inner hair cell, OHC outer hair cell, VHC vestibular hair cell, max projection, maximum intensity projection. All error bars show standard deviation.

To study the role of KLHDC7B in the auditory system we first investigated its expression in the mouse inner ear and several other tissues. Like the human *KLHDC7B* gene, the mouse *Klhdc7b* gene encodes two putative isoforms that differ only in which methionine is annotated as the predicted start codon. It is possible to specifically and individually detect the long isoform transcript [Ensembl transcript ENST00000648057.3], but not the short isoform transcript [Ensembl transcript ENST00000395676.4] since its sequence is common to both transcripts. We therefore designed two sets of qPCR primers and probes, one hybridizing to the unique long portion of the transcript only (L), and the other probe hybridizing within the region common to both long and short isoforms (L + S) (Fig. 1A, top). qPCR data shown in Fig. 1 are normalized as ΔCT relative to expression of the housekeeping gene *Drosha*, then represented as Relative Quantification (RQ) to adult kidney (A) or P1 kidney (B) due to its low expression level in kidney*. Klhdc7b* is expressed at relatively high levels in adult mouse cochlea compared with other tissues, significantly higher than in eye, kidney, stomach and brain tissue for the combined long and short isoform assay (p < 0.0002, (Fig. 1A). To assess the expression level of *Klhdc7b* in mouse cochlea over time, we collected cochleae at postnatal days 1, 7, in adult, and aged animals, and confirmed that expression is relatively high throughout life in cochlea, with highest expression in adult cochlea which was significantly higher than in other ages tested (p < 0.02, Fig. 1B).

### KLHDC7B is expressed in hair cells

After determining that *Klhdc7b* is expressed in the cochlea via qPCR, we aimed to pinpoint which specific cell types within the inner ear express *Klhdc7b* using in situ hybridization and immunofluorescent staining. As in the qPCR probe design (Fig. 1A), two RNAscope probes were designed to detect the common portions of both transcripts (L + S) or a portion unique to the long (L) *Klhdc7b* transcript. We performed RNAscope in conjunction with immunostaining for Myosin VIIa (MYO7A), a hair cell marker (Fig. 1C). The L + S probe detected transcripts exclusively within hair cells, the sensory cells of the cochlea (Fig. 1C) and vestibular system (Fig. 1D); inner, outer, and vestibular hair cells (IHC, OHC, VHC in Fig. 1C, D) and were not observed in other cell types. The long isoform-specific probe indicates a very similar pattern of hair cell expression although some background stain can be seen outside of the hair cell region in the Organ of Corti (Fig. 1C, D). Whole cochleae are presented in Supplemental Fig. 1. Furthermore, immunostaining with a custom-generated antibody to KLHDC7B (the immunogen was the entire short human isoform, which has high sequence homology to mouse KLHDC7B) is also observed to be exclusively in inner and outer hair cells within the cochlea (Fig. 1E, F; Suppl. Fig. 1). Staining within OHCs localizes predominantly to the plasma membrane, as determined by a co-stain with prestin, a protein localized to the outer hair cell membrane (Fig. 1E, G, H, Suppl. Fig. 2). Staining within IHCs appears less intense than in OHCs. It also appears to localize to the plasma membrane in these cells, but unlike OHCs it is also present in the cytoplasm in these cells (Fig. 1F).

### Generation of * Klhdc7b* knockout mice

**Fig. 2:**
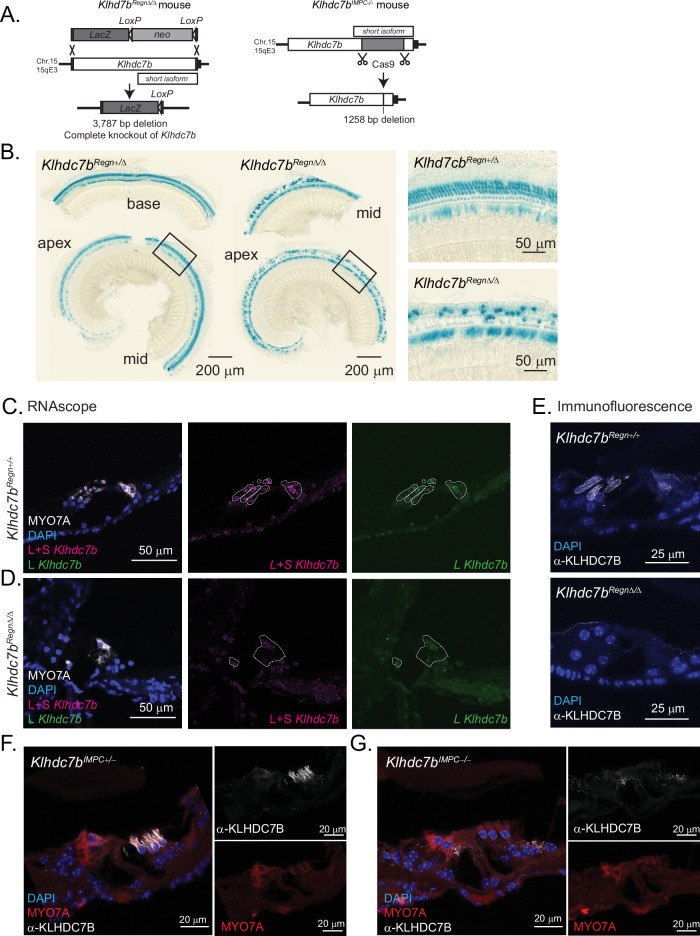
Mutant mouse model design and validation. **A** Left, schematic showing knockout design for *Klhdc7b*^*Regn*Δ/Δ^ mice. The whole *Klhdc7b* gene was excised from the mouse genome and replaced with a beta-galatosidase (LacZ) expression cassette for visualization. Right, knockout design for *Klhdc7b*^*IMPC-/-*^ mice. CRISPR/Cas9 was used to create a 1258 base pair deletion creating a truncating premature stop codon and a null allele. The location of the putative short isoform is shown with a smaller box above or below the *Klhdc7b* long isoform in both schematics. **B** LacZ expression in wholemount preparations of the organ of Corti of heterozygous (*Klhdc7b*^*Regn*+/Δ^) and knockout (*Klhdc7b*^*Regn*Δ/Δ^) mice (males age 10 weeks). Some OHC loss **C** 40x confocal images showing RNAscope *Klhdc7b* probes for the L and L + S transcripts and hair cell marker MYO7A immunofluorescence of *Klhdc7b*^*Regn+/+*^ mouse organ of Corti. DAPI stains nuclei. Outlines of MYO7A staining are shown overlaid in white on individual probe channels to delineate hair cells. **D** As **C**, but in *Klhdc7b*^*Regn*Δ/Δ^ mouse organ of Corti. For **C**, **D**, mice are females age 8 weeks. **E** KLHDC7B immunofluorescence of *Klhdc7b*^*Regn+/+*^(top) and *Klhdc7b*^*Regn*Δ/Δ^ mice (bottom). Mice are 13–14 weeks, WT is male and KO is female. **F** Confocal images showing cochleae from *Klhdc7b*^*IMPC+/-*^ mouse organ of Corti at postnatal day 19. Sections have been stained for KLHDC7B (white), MYO7A (red) and DAPI (blue) (female postnatal day 19). **G** Same as **F**, in *Klhdc7b*^*IMPC-/-*^ mouse organ of Corti (female at postnatal day 19).

To investigate the functional role of *Klhdc7b* in the cochlea and to determine whether KLHDC7B is required for hearing, two transgenic mouse lines were generated, *Klhdc7b*^*Regn*Δ/Δ^ and *Klhdc7b*^*IMPC-/-*^, designed to knock out both the putative long and short isoforms of the gene on different genetic backgrounds. The predominant strain used in transgenic mouse models is the C57BL/6 N strain which contains a strain-specific allele (*Cdh23*^*ahl*^) that confers a progressive age-related hearing loss on these mice^23^. *Klhdc7b*^*IMPC-/-*^mice were generated on the C57BL/6 N background (containing the age-related hearing loss mutation in *Cdh23*) as part of the International Mouse Phenotyping Consortium^24,25^ by clustered regularly interspaced short palindromic repeat (CRISPR)-induced mutation (Fig. 2A, right). This created a 1258 nucleotide deletion within the single exon introducing a premature stop codon and a null allele^26^. *Klhdc7b*^*Regn*Δ/Δ^ were generated on the B6.CAST-*Cdh23*^*753A>G*^ background with a 3787 bp deletion encompassing both the putative long and short isoforms of the gene (Fig. 2A, left).

Mice for both lines were born at expected Mendelian ratios and appeared phenotypically normal. Since a *LacZ* reporter cassette was inserted within the coding region of *Klhdc7b* in the *Klhdc7b*^*Regn*Δ/Δ^ line, we identified *Klhdc7b* expressing cells using Beta galactosidase (Fig. 2B). At 10 weeks of age, LacZ can be observed in the single row of IHCs and three rows of OHCs in cochlear epithelia prepared from *Klhdc7b*^*Regn*Δ/Δ^ and *Klhdc7b*^*Regn*+/Δ^ mice (Fig. 2B). Patchy LacZ stain in the 3 OHC rows represents missing OHCs, further investigated in later figures, that can already be observed at this age in *Klhdc7b*^*Regn*Δ/Δ^ mice. No other cells in the inner ear, including vestibular hair cells, showed LacZ staining and no other tissues, including brain and liver, clearly expressed LacZ, which was somewhat surprising since we detected *Klhdc7b* expression in liver and brain cells and vestibular hair cells using RNA-based techniques (Fig. 1A). Immunostaining of vestibular hair cells also suggests KLHDC7B (Suppl. Fig. 1A, B) is absent, suggesting that the LacZ staining reflects protein expression. One possible explanation for the discrepancy in RNA and protein data is that the shorter *Klhdc7b* transcript may not be translated into protein.

Importantly, using both the custom anti-KLHDC7B antibody and RNAScope probes no signal was detected in hair cells of *Klhdc7b*^*Regn*Δ/Δ^ mice (Fig. 2C–E). *Klhdc7b*^*IMPC-/-*^ mice also showed no presence of anti-KLHDC7B immunostaining in hair cells whereas in *Klhdc7b*^*IMPC+/-*^ mice immunostaining is localized to the membrane of inner and outer hair cells (Fig. 2F, G) similar to that observed in wildtype mice. (Fig. 2E). Once again staining in the IHC of wildtype and heterozygote mice appears less intense than that in OHCs.

To examine a role for *Klhdc7b* in the central nervous system, we performed a suite of behavioral tests to determine whether there were any changes in exploratory behavior (open field test), spatial working memory (Y-Maze), or motor function and balance (pole test, rotarod). We confirmed that both sexes of *Klhdc7b*^*Regn*^^+/+^ and *Klhdc7b*^*Regn*Δ/Δ^ mice performed indistinguishably on all four tests (Suppl. Fig. 3). Other measures of phenotypic differences, including weight, bone volume and fat volume, spleen immune profiling, blood immune cell profiling, and serum chemistry were all normal. *Klhdc7b*^*IMPC-/-*^ mice underwent wide-ranging phenotype and behavioral testing as part of the IMPC program, the results of which are available at https://www.mousephenotype.org/data/genes/MGI:3648212. At the start of this study hearing associated abnormalities were the only significant phenotypes recorded in *Klhdc7b*^*IMPC-/-*^ mice. More recently, data released by the IMPC suggest evidence of new additional phenotypes. These include increased eosinophil cell number and decreased lymphocyte cell number in female *Klhdc7b*^*IMPC-/-*^ mice in late adulthood. We included both sexes in our experiments and did not observe any apparent differences between males and females in either mouse model although as a result of using litter mates it is not well-powered enough per sex per genotype at each time point to perform statistical tests.

### Both *Klhdc7b* knockout mouse models show early onset progressive hearing loss

**Fig. 3:**
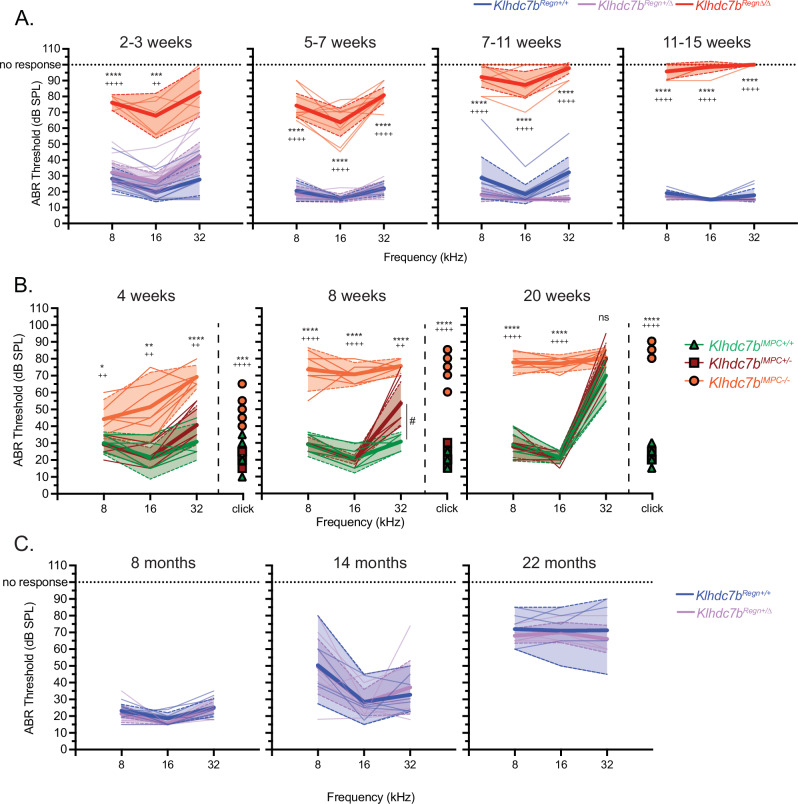
*Klhdc7b-*deficient mice exhibit early onset progressive hearing loss. **A** ABRs were recorded in separate cohorts of *Klhdc7b*^*Regn+/+*^, *Klhdc7b*^*Regn*+/Δ^ and *Klhdc7b*^*Regn*Δ/Δ^ mice at four time points. 100 dB denotes no response. n = 3–17 mice per group, mixed sex. **B** ABRs were recorded longitudinally in a cohort of *Klhdc7b*^*IMPC+/+*^*, Klhdc7b*^*IMPC+/-*^ and *Klhdc7b*^*IMPC-/-*^ mice at three time points. n = 6–7 mice per group, mixed sex. **C** ABRs were recorded longitudinally in a cohort of *Klhdc7b*^*Regn+/+*^, *Klhdc7b*^*Regn*+/Δ^ mice at three older time points. n = 5–8 mice per group, mixed sex. For **A**–**C**, mean ABR (±SD) thresholds for click and pure-tone response are shown. Analysis was performed via two-way ANOVA, using Tukey’s test for post-hoc comparisons. For A–**C** *p < 0.05, **p < 0.01, ***p < 0.001, ****p < 0.0001 between knockout (*Klhdc7b*^*Regn*Δ/Δ^*, Klhdc7b*^*IMPC-/-*^*)* compared with wild type (*Klhdc7b*^*Regn+/+*^, *IMPC*^*+/+*^) mice. + p < 0.05, ++ p < 0.01, +++ p < 0.001, ^*++*^++ p < 0.0001 between knockout and heterozygous (*Klhdc7b*^*Regn*+/Δ^*, Klhdc7b*^*IMPC+/-*^*)* mice. # p < 0.05 between wild type and heterozygous mice. For **A**–**C**, thicker lines indicate means, thin lines individual mice, and shaded areas between dotted lines are 95% confidence intervals.

Given its specific expression in cochlear hair cells, we studied the role of KLHDC7B in the inner ear, by measuring Auditory Brainstem Responses (ABR) in wildtype, heterozygous and homozygous mutant mice for both *Klhdc7b*^*Regn*Δ/Δ^ (Fig. 3A) and *Klhdc7b*^*IMPC-/-*^ (Fig. 3B) knockout models at several ages. ABRs measure the electrical activity of the auditory pathway in response to a sound stimulus as the electrical signal travels from the cochlear nerve up through the brainstem toward the auditory cortex. Hearing function in mice begins at two weeks of age. At postnatal day 17 (P17, 2–3 weeks), just after the onset of hearing, *Klhdc7b*^*Regn*Δ/Δ^ mice exhibit significantly elevated ABR thresholds compared to their littermates (Fig. 3A, 2–3 weeks), indicating a severe hearing loss. Additionally, the *Klhdc7b*^*Regn*Δ/Δ^ mice have a significantly diminished Wave 1 amplitude compared to their littermates (Supplementary Fig. 4), likely reflecting differences in thresholds, particularly since there were no major differences in Wave I latency (Supplementary Fig. 4). ABR thresholds become progressively elevated as the *Klhdc7b*^*Regn*Δ/Δ^ mice age, such that most have no response at any of the three tested frequencies by 11-15 weeks of age (Fig. 3A).

Similarly, *Klhdc7b*^*IMPC-/-*^ mice exhibit elevated ABR thresholds at 4 weeks of age, the youngest age tested, showing moderate hearing loss at lower frequencies progressing to severe at 32 kilohertz (kHz), the highest frequency tested (Fig. 3B). In *Klhdc7b*^*IMPC-/-*^ mice, hearing loss progresses with age reaching a ~ 80 decibel sound pressure level (dB SPL) threshold by 20 weeks of age across all frequencies tested. At this age the effect of the *Cdh23*^*ahl*^ allele in the C57BL/6 N strain is becoming evident, with significantly elevated high-frequency (32 kHz) ABR thresholds also observed in heterozygote and wildtype mice (Fig. 3B).

For both *Klhdc7b* knockout models there was no overt hearing loss phenotype in heterozygous mice although small significant elevation of thresholds compared to wildtype mice were found only at 32 kHz at some time points (Fig. 3A left, 3B middle). To test whether there might be an age-related phenotype in heterozygous mice, *Klhdc7b*^*Regn*+/Δ^ mice were aged along with *Klhdc7b*^*Regn*^^+/+^ controls. ABR thresholds (Fig. 3C) and Wave 1 amplitude and latency (Supplementary Fig. 5) were indistinguishable from *Klhdc7b*^*Regn*+/+^ mice as late as 22 months of age, although both genotypes had diminished amplitudes at later ages (Supplementary Fig. 5). Interestingly, heterozygous *IMPC*^*+/-*^ mice develop high-frequency hearing loss at 32 kHz earlier than their wildtype counterparts, having higher thresholds at 8 weeks of age (Fig. 3B, middle).

**Fig. 4:**
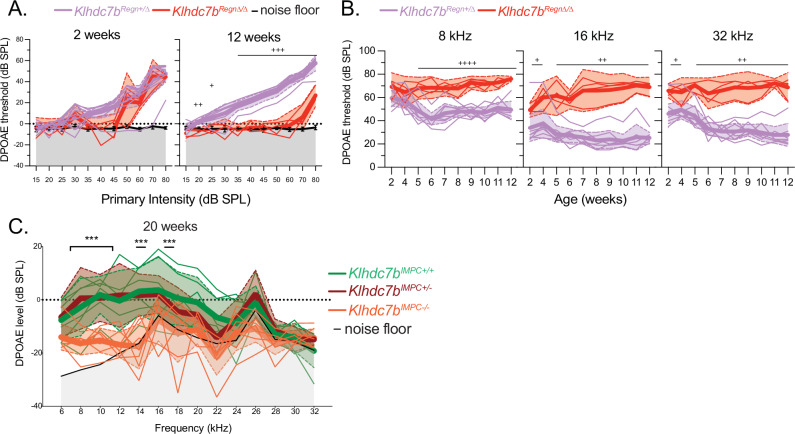
DPOAE in *Klhdc7b-*deficient mice indicate outer hair cell dysfunction. **A**, **B** DPOAE longitudinally recorded in *Klhdc7b*^*Regn+/+*^, *Klhdc7b*^*Regn*+/Δ^ and *Klhdc7b*^*Regn*Δ/Δ^ mice. **A** Input-output curves of DPOAE signal vs. primary stimulus intensity at 2 and 12 weeks for 16 kHz. **B** DPOAE thresholds over time at three frequencies. n = 4–9 mice per group. **C** DPOAEs recorded in *Klhdc7b*^*IMPC+/+*^*, Klhdc7b*^*IMPC+/-*^, and *Klhdc7b*^*IMPC-/-*^ mice plotted against frequency. The stimul**i** were presented in 2 kHz intervals at 65/55 dB SPL for the f1 and f2 tones, respectively. For **A**–**C** data were analyzed via two-way ANOVA followed by Tukey’s test (**B**, 8 kHz) or unpaired t tests (**C**). If data failed normality (**A**, **B** 8 kHz and 16 kHz) testing they were analyzed via Mann–Whitney tests. *p < 0.05, **p < 0.01, ***p < 0.001, ****p < 0.0001 between knockout (*Klhdc7b*^*Regn*Δ/Δ^*, Klhdc7b*^*IMPC-/-*^*)* and wild type (*Klhdc7b*^*Regn+/+*^, *Klhdc7b*^*IMPC+/+*^*)* mice. + p < 0.05, ++ p < 0.01, +++ p < 0.001, ++*++* p < 0.0001 between knockout and heterozygous (*Klhdc7b*^*Regn*+/Δ^*, Klhdc7b*^*IMPC+/-*^) mice. Thicker lines indicate means, thin lines individual mice, and shaded areas between dotted lines are 95% confidence intervals.

To further characterize the hearing phenotype of these mice, we recorded DPOAE to assess the in vivo function of outer hair cells and found that there was remaining OHC function in *Klhdc7b*^*Regn*Δ/Δ^ mice at 2–3 weeks, with very little remaining by 12 weeks when the ABR signal has disappeared (Fig. 4A, right). To better understand the time course of the loss of OHC function, a cohort of *Klhdc7b*^*Regn*Δ/Δ^ and *Klhdc7b*^*Regn*+/Δ^ littermates were longitudinally recorded every week from 2–3 weeks to 12 weeks of age. There is some remaining OHC function at 2–3 weeks of age, with DPOAE thresholds becoming significantly elevated in *Klhdc7b*^*Regn*Δ/Δ^ mice compared with *Klhdc7b*^*Regn*+/Δ^ from 5 weeks of age (Fig. 4B). When tested at 20 weeks of age, *Klhdc7b*^*IMPC-/-*^ mice exhibit a similar loss of OHC function, as observed in *Klhdc7b*^*Regn*Δ/Δ^ mice, whereas their wildtype and heterozygous littermates continue to show good low frequency DPOAE responses (Fig. 4C).

### Outer hair cells in both *Klhdc7b* knockout mouse models develop normally, but begin to die around hearing onset

**Fig. 5:**
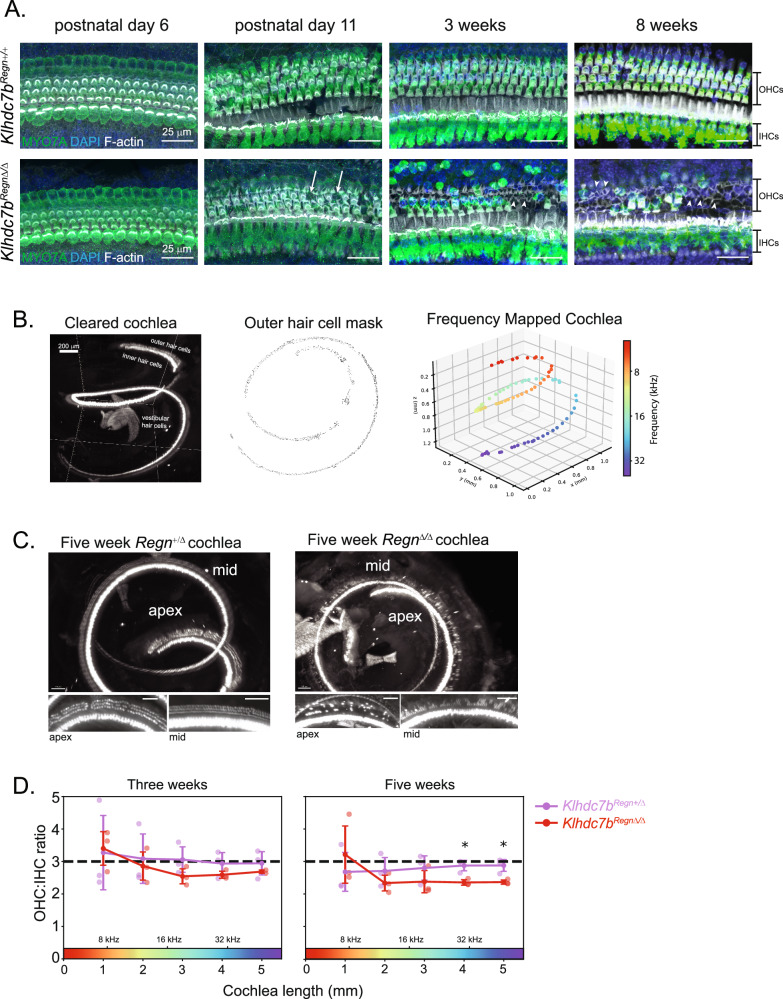
Outer hair cell death in *Klhdc7b*^*Regn*Δ/Δ^ mice. **A** Immunostaining of whole mounted cochlea of *Klhdc7b*^*Regn*^^+/+^, and *Klhdc7b*^*Regn*Δ/Δ^ mice at postnatal day (p) 6, p11, 3 weeks and 8 weeks of age. MYO7A (green) specifically labels hair cells, DAPI (blue) stains nuclei, F-actin (white) labels stereocilia. Arrows denote missing OHCs. Arrowheads denote examples of supporting cell scars where OHCs have died. Asterisks denote rounded MYO7A staining of cells outside the normal hair cell location. **B** Cochleae were stained for MYO7A and a nuclear stain, then cleared and imaged using light-sheet microscopy. For one example cochlea, Left, MYO7A staining in 3D. Middle, automatically generated outer hair cell mask. Right, frequency mapping along the tonotopic axis from apex to base. **C** Representative example images of *Klhdc7b*^*Regn*+/Δ^ (left) and *Klhdc7b*^*Regn*Δ/Δ^ (right) cochleae at five weeks visualized from the top down. Below each image of intact cochlea are example images of apex (left) and middle (right) turns. **D** Outer to inner hair cell ratios at three weeks and five weeks from *Klhdc7b*^*Regn*Δ/+^ and *Klhdc7b*^*Regn*Δ/Δ^cochleae. n = 3 mice per genotype, two-tailed Welch’s t-test, *p < 0.05, all error bars show standard deviation.

The hearing phenotype of *Klhdc7b*^*Regn*Δ/Δ^ and *Klhdc7b*^*IMPC-/-*^ mice led us to examine the anatomy of the organ of Corti in these mice. Examples of middle-apical turn of the organ of Corti of *Klhdc7b*^*Regn*Δ/Δ^ and *Klhdc7b*^*Regn*^^+/+^ at different ages are shown in Fig. 5A. At P6, the gross anatomy of the organ of Corti of *Klhdc7b*^*Regn*Δ/Δ^ mice appear normal, immunostaining with hair cell marker MYO7A shows that the 3 rows of outer hair cells and single row of inner hair cells are intact and stereocilia are present, stained with F-actin, (shown in examples from the middle turn in Fig. 5A). However, by P11 a few MYO7A+ outer hair cells appear to be missing (Fig. 5A, white arrows). By 3 weeks, many outer hair cells are missing and there are visible supporting cell scars (Fig. 5A, white arrowheads), which are known to form when hair cells die^27^. Circular stained areas of MYO7A (Fig. 5A, asterisks) are present beyond the normal border of the hair cell location, which may suggest engulfment of dead hair cells by supporting cells^28,29^. By 8 weeks of age, almost no outer hair cells are present and evident scarring is visible as a lattice-like pattern of F-actin staining (Fig. 5A, arrowheads). This loss of outer hair cells is consistent with the progressive loss in hearing detected by ABR and the reduction in DPOAE levels.

Additionally, we quantified hair cells in intact cochlea using light-sheet microscopy to better visualize all regions of the cochlea. This method has been previously published in gerbil^30^. We adapted the protocol for mouse and utilized the anti-MYO7A antibody and light-sheet microscopy to visualize hair cells (Fig. 5B). Inner and outer hair cell masks were automatically annotated and projected onto the frequency map of the mouse cochlea. A clear reduction in outer hair cells was apparent at 5 weeks (Fig. 5C). At three weeks of age, the ratio of outer to inner hair cells along the length of the cochlea is slightly smaller in *Klhdc7b*^*Regn*Δ/Δ^ than in *Klhdc7b*^*Regn*+/Δ^ mice, and the difference is significant toward the base of the cochlea at five weeks of age (Fig. 5D).

**Fig. 6:**
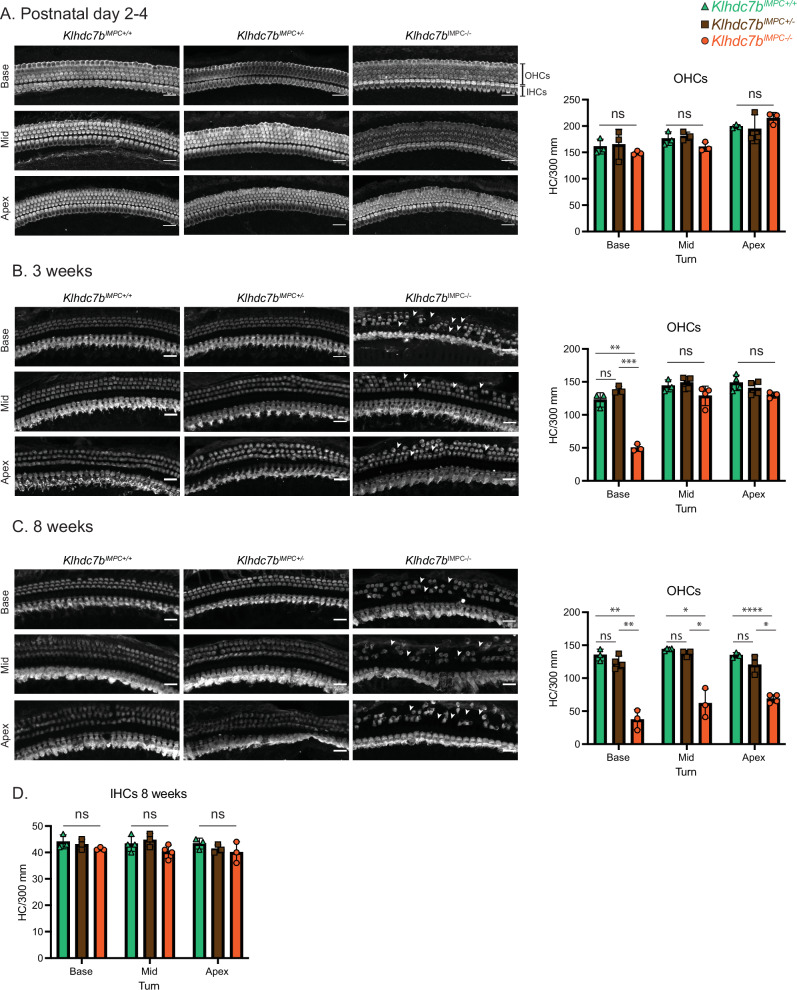
Progressive outer hair cell loss in *Klhdc7b*^*IMPC-/-*^ mice is evident from 3 weeks of age. **A**–**C** Immunofluorescence in wholemount cochlea preparations using MYO7A to label hair cells in *Klhdc7b*^*IMPC+/+*^*, Klhdc7b*^*IMPC+/-*^ and *Klhdc7b*^*IMPC-/-*^ mice. Panels show (x20) representative confocal microscopy images from all three genotypes. Charts to the right show OHC quantification at each cochlea turn, base, mid and apical. Scale bar = 5 µm. Data is shown for 3 ages: **A** postnatal day 2–4; **B** 3 weeks and at 8 weeks of age (**C**). Arrowheads show examples of missing outer hair cells. Sex of animals in images (**A**) was not assigned in neonatal animals. **B** From left to right, *base:* female/male/female; *mid*: female/ male/ female; *apex*: female/male/female. **C** From left to right, *base:* female/female/male; *mid*: male/ male/ male; *apex*: female/male/male. **D** Chart shows quantification of inner hair cells (IHCs) at 8 weeks of age. Statistical analysis was performed via one-way ANOVA, using Tukey’s test for post-hoc comparisons, ns not significant, *p < 0.05, **p < 0.01, ***p < 0.001, ****p < 0.0001, all error bars show standard deviation.

The *Klhdc7b*^*IMPC-/-*^ mouse model also showed a similar progressive outer hair loss (Fig. 6 and Supplementary Fig. 3). At early postnatal time points (P2, Fig. 6A), staining for MYO7A reveals both the 3 rows of outer hair cells and single row of inner hair cells appeared normal with no significant difference in hair cell number between all genotypes. At 3 weeks, there was significant outer hair cell loss at the base of the cochlea (Fig. 6B), but not in the mid or apical turns. By 8 weeks old this had progressed to significant outer hair cell loss at all portions of the cochlea, with no inner hair cell loss at this age (Fig. 6C, D). The timing of outer hair cell death corresponded to the progressive hearing loss shown in Fig. 3 for both models. Supplementary Fig. 6 shows higher resolution images with immunostaining of supporting cell marker Sox2 and F-actin labeled stereocilia in which morphology of the non-sensory epithelium and stereocilia appear similar in *Klhdc7b*^*IMPC-/-*^*, Klhdc7b*^*IMPC+/-*^ and *Klhdc7b*^*IMPC+/+*^at early ages.

### Outer hair cells appear to develop normal stereocilia and functional mechanotransduction channels

**Fig. 7:**
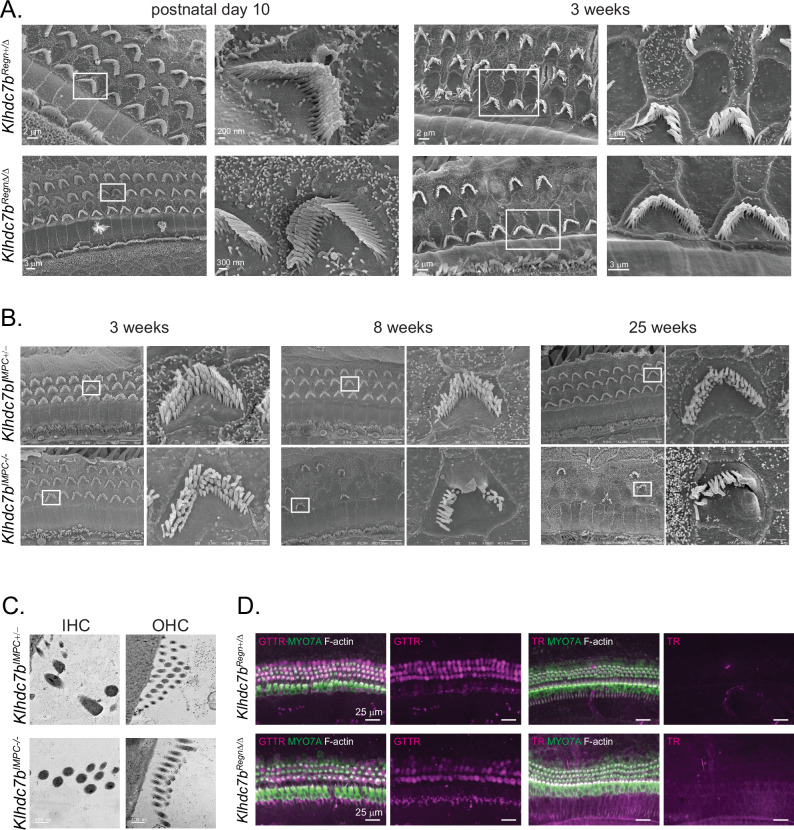
Stereocilia and mechanotransduction channel function appear normal in *Klhdc7b-*deficient mice. **A** SEM of *Klhdc7b*^*Regn*Δ/+^ and *Klhdc7b*^*Regn*Δ/Δ^cochlea at P10 and 3 weeks. Stereocilia appear grossly normal, even after significant hair cell loss at 3 weeks. **B** SEM of *Klhdc7b*^*IMPC+/-*^ and *Klhdc7b*^*IMPC-/-*^ cochlea at 3 weeks (left, male mouse), 8 weeks (middle, male mouse), and 25 weeks (right, female mouse) of age show normal development of stereocilia. Scale bar in left images at each time point, 10 μm, in cutout images 1 μm. **C** TEM of stereocilia of *Klhdc7b*^*IMPC+/-*^, and *Klhdc7b*^*IMPC-/-*^ cochlea at 3 weeks suggest IHCs and OHCs have normal stereocilia. **D** Cochlear explant cultures of *Klhdc7b*^*Regn*Δ/+^ and *Klhdc7b*^*Regn*Δ/Δ^ mice treated with Gentamycin-Texas Red (GtTR, left) or Texas Red (TR, right) alone, then immunostained for MYO7A labeling hair cells and stained for F-actin (stereocilia). Entry of GtTR into hair cells confirms mechano-transduction in both *Klhdc7b*^*Regn*Δ/+^ and *Klhdc7b*^*Regn*Δ/Δ^ mice.

Deficits in stereocilia formation or morphological abnormalities can lead to deafness and hair cell death^31–35^. To determine whether the hair cell degeneration was driven by stereocilia abnormalities, we performed SEM and TEM in organ of Corti from our mutant mouse models. SEM in *Klhdc7b*^*Regn*+/Δ^ and *Klhdc7b*^*Regn*Δ/Δ^ mice at postnatal days 10 (P10) and 3 weeks were undertaken to visualize the organ of Corti at time points before and during outer hair cell degeneration. Sample images from the middle turn of the organ of Corti are pictured in Fig. 7A. Both *Klhdc7b*^*Regn*+/Δ^ mice and *Klhdc7b*^*Regn*Δ/Δ^ mice have stereocilia that appear grossly normal at P10 (Fig. 7A, left). By 3 weeks, many outer hair cells are absent, however, the stereocilia bundles on the remaining outer hair cells are intact with apparently normal morphology (Fig. 7A, right). Similarly, SEM data demonstrated the stereocilia bundle of most OHC appears normal in *Klhdc7b*^*IMPC-/-*^ mice at 3 weeks of age (Fig. 7B, left). By 8 weeks and 25 weeks of age some stereocilia pathology is visible in surviving outer hair cells of *Klhdc7b*^*IMPC-/-*^ mice, potentially due to hair cells being in the process of dying (Fig. 7B, middle and right). Transmission electron microscope (TEM) images also suggest IHC and OHC stereocilia appear normal in mice at 3 weeks of age (Fig. 7C). These data suggest that KLHDC7B is not critical for the development and integrity of the stereocilia bundle.

Mechanotransduction (MET) defects can also cause hair cell death^36^, so we sought to assess MET complex function using a gentamycin-Texas red assay (GtTR). Gentamycin, like other aminoglycosides, is known to readily enter hair cells via the MET complex and this property is maintained when conjugated to a fluorescent dye, Texas red^37–40^. In the absence of functional MET complexes GtTR will not enter hair cells thus allowing us to assess hair cell function before the onset of hearing in mice. To evaluate this, we cultured neonatal organ of Corti from *Klhdc7b*^*Regn*Δ/Δ^ and *Klhdc7b*^*Regn*^^+/+^ mice at P4-P5 for 3 days before incubating with GtTR for 20 min. GtTR labeling was observed in hair cells for both the *Klhdc7b*^*Regn*Δ/Δ^ and *Klhdc7b*^*Regn*^^+/+^ cultures, indicating that MET complexes are assembled and functional (Fig. 7D).

At early time points in *Klhdc7b*^*Regn*Δ/Δ^ mice when DPOAEs are relatively normal the ABR threshold is elevated more than might be expected due to an OHC deficit alone. To investigate further we recorded a cohort of 3 week old *Klhdc7b*^*Regn*Δ/Δ^*, Klhdc7b*^*Regn*+/Δ^ and *Klhdc7b*^*Regn*^^+/+^ mice and stained for markers of synaptic punctae (CTBP2 to label IHC synaptic ribbons and GLUA2 to label postsynaptic AMPA receptors) to determine whether there was a possible synaptic dysfunction^41^. ABR thresholds were significantly elevated in *Klhdc7b*^*Regn*Δ/Δ^ mice above the expected 25–50 decibels (dB) that would likely be due to an OHC deficit alone^42^. However, wave I latencies were not significantly different between genotypes, and synaptic punctae were identical between all three genotypes, suggesting a possible IHC deficit but not one caused by a synaptic phenotype.

## Discussion

The auditory system is highly complex, encompassing multiple tissues and many specialized cell types. This is evidenced from the high heterogeneity of functions and cell-specific expression of genes associated with monogenic hearing loss, with several hundred loci found to date^43^, accessed 20 Sept 2024. Although good progress has been made in understanding monogenic hearing loss, the genes and hence pathogenic mechanisms that underlie the highly prevalent ARHL have been much more difficult to identify.

Early GWAS with well-characterized ARHL cohorts largely did not identify genome-wide significant associations^6^. Recent GWAS with much larger cohorts have been more successful, identifying over 50 associations, but these have largely relied on self-report hearing data or medical records rather than more reliable audiometric measures to define the phenotype. Therefore, some doubt remained about whether these associations represent true ARHL susceptibility genes. Our results here demonstrate that *Klhdc7b*, the ortholog of a novel gene strongly associated with hearing loss in several recent human GWAS focused on age-related hearing loss, is necessary for the maintenance of hearing in mice. This provides a strong corroboration of the findings of recent GWAS using self-reported hearing loss data.

Our data suggest that *Klhdc7b* is not necessary for the development and functional maturation of hair cells, as the sensory epithelium appears to be normal at early postnatal time points before the onset of hearing (Figs. 5, 6). This includes the arrangement of their stereocilia and the assembly of functional mechanotransduction complexes (Fig. 7). The observation that the *Klhdc7b*^*IMPC-/-*^ model still retains some hearing particularly at lower frequencies at 1 month of age as well as remaining DPOAE at early ages in the *Klhdc7b*^*Regn*Δ/Δ^ model (Fig.4) also suggests that the hair cells that develop are functional. Taken together, these results suggest that *Klhdc7b* is required for maintenance and survival of outer hair cells.

The length and stiffness of outer hair cells varies along the length of the cochlea, allowing outer hair cells to be frequency-selective^44^. Outer hair cells are known to be more sensitive than inner hair cells, dying earlier than inner hair cells in response to insults such as drug-induced toxicity and noise exposure^45–47^. This generally occurs in a base-to-apex pattern, perhaps due to differences in the properties of hair cells or differential exposure to stress along the length of the cochlea. This base-to-apex pattern is observed in early stages of ARHL, later progressing to inner hair cell loss^42,48,49^. Both *Klhdc7b* knockout models exhibit a base-to-apex pattern of outer hair cell loss (Figs. 5, 6). The causes of outer hair cell death are known to be diverse; deficits in electromotility^50^, specific calcium buffering proteins^51,52^, autophagy^53^, and ER stress regulation^54^ can all cause outer hair cell death and hearing loss. Therefore, the requirement of *Klhdc7b* for the survival of outer hair cells does not necessarily provide clues as to its function.

While KLHDC7B does not appear to be required for the survival of inner hair cells our data do not rule out a functional inner hair cell deficit. Arguably, ABR thresholds are elevated beyond what might be expected for an OHC-specific effect, especially at early time points when DPOAEs suggest some OHC function is retained (Fig. 3 and Supplementary Fig.7). However, other mouse models that have specific outer hair cell dysfunction or loss show decreases in ABR thresholds that are consistent with our model^50,55,56^. According to our data, KLHDC7B is expressed in inner hair cells, so presumably it has some role in these cells, although levels of IHC anti-KLHDC7B stain appear lower and with a less membrane-specific localization than OHCs (Figs. 1, 2). Perhaps KLHDC7B performs a different function in IHCs than OHCs, or a similar function that is more critical for outer hair cell survival.

Although we have confirmed the necessity of KLHDC7B for maintenance of auditory function we have not identified the molecular function of KLHDC7B in the inner ear. There is very little published information on the role and molecular function of KLHDC7B. We show that KLHDC7B appears to be localized to or near the plasma membrane of hair cells, with stronger staining observed in outer hair cells. While other members of the Kelch domain-containing superfamily have diverse cellular locations and functions^20,21^, some do bind to actin, and many are involved in cell structure and organization by acting as scaffolds for protein-protein interactions^20^. KLHDC7B also has a predicted transmembrane domain close to the N terminus of the putative long isoform of both the mouse and human protein (amino acids 26–47 in both) according to Phobius, a web-based protein domain prediction tool^57,58^.

The structure near the plasma membrane in outer hair cells is complex. Just inside the plasma membrane of outer hair cells are structures called the cortical lattice (CL) and subsurface cisternae (SSC). The cortical lattice links the subsurface cisternae with the plasma membrane, and it consists of actin molecules cross-linked by spectrin beta V. There are two unknown structural components in this region, the “pillar” linking the cortical lattice with the plasma membrane, and the actin- subsurface cisterna connection that links the actin within the CL to the SSC^59^. Further research is needed to determine whether KLHDC7B could be involved in these structures or related to their function.

The two *Klhdc7b* knockout models were generated using independent strategies and on different mouse strain backgrounds (Fig. 2). Despite this, the phenotypes of both knockout lines are remarkably consistent although with some minor differences. Surprisingly, the hearing deficit in the *Klhdc7b*^*IMPC-/-*^ mice on the *Cdh23*^*ahl*^ background is less severe and has a slightly later onset than that found in the *Klhdc7b*^*Regn*Δ/Δ^ mice on the B6.CAST.*Cdh23*^*753A>G*^ background (Fig. 3). This suggests that there is no evidence of a genetic interaction between the *Cdh23*^*ahl*^ allele and the *Klhdc7b* knockout to produce an earlier onset phenotype as has been found in other hearing gene models^54,60–62^. It is possible that variations in the conditions of animal housing environments (e.g., noise), and ABR measurements between the two host organizations might account for these minor differences.

In both models, loss of *Klhdc7b* in mice resulted in an early onset and fast progressing hearing loss, whereas data from human GWAS has suggested that variation in *KLHDC7B* is associated with increased risk of ARHL, a much later onset phenotype. This may be accounted for by the different effects of a null allele compared to those of a missense variant detected in the GWAS. However, the requirement of KLHDC7B for survival rather than development of outer hair cells is consistent with the common pathology of ARHL, where progressive outer hair cell loss occurs before inner hair cells^63^.

The lack of a phenotype in heterozygous *Klhdc7b*^*Regn*+/Δ^ mice suggests that loss of one copy of the *Klhdc7b* gene is not deleterious to outer hair cell survival in mice. This is of interest since some rare heterozygous loss of function variants in KLHDC7B have been associated with ARHL in human GWAS^18^. However, a heterozygous mutation that results in hair cell degeneration over the course of decades in humans exposed to noise and other ototoxic insults may not present within the 2.5-year lifespan of the mouse without the same environmental exposures. It would be interesting to test whether *Klhdc7b*^*Regn*+/Δ^ mice are more susceptible to outer hair cell loss in response to an insult such as noise exposure. *Klhdc7b* expression has been shown to increase in outer hair cells after noise-induced hearing loss^64^, which could be a compensatory response to injury.

The variant in *KLHDC7B* which has been consistently and strongly associated with ARHL in recent GWAS is a missense variant which causes a valine to methionine change in both long and short forms of KLHDC7B^16,18,65,66^. Interestingly, both Polyphen and Sift predictions suggest that this variant would be deleterious to the long version of the protein but not to the short isoform (Supplementary Table 1). Protein modeling (Supplementary Fig. 8) indicates that the additional alpha helices in the longer form of KLHDC7B may interact and alter the β-propeller structure common to both long and short forms suggesting the two isoforms might have subtly distinct β-propellers. Missense 3D^67^ also indicates that the 1154Val>Met change in the long form is structurally damaging (SupplementaryFig. 9). Our expression studies show that the longer form of KLHDC7B is expressed in hair cells in mice. The 1154 valine amino acid is conserved in the mouse gene meaning it should be possible to create a knock-in mouse model of the human Val>Met mutation to investigate the effect on outer hair cell survival.

Overall, this work supports the use of large-scale GWAS studies to identify novel genetic associations with hearing loss, even when these studies do not have comprehensive auditory phenotyping. Our results translate a finding from a genome-wide association study in humans back into mice, allowing us to fully study the function of the gene in a controlled manner in a model system where the inner ear is much more accessible than in humans. We were able to identify the cellular expression pattern, subcellular location, and characterize the phenotype in two knockout mouse models of *Klhdc7b*. The very similar phenotype in two mouse models generated and analyzed at two different institutions and mouse facilities, possibly with different noise levels, on different backgrounds with respect to a known age-related hearing loss mutation, strengthens the case for a specific hearing loss phenotype caused by the loss of KLHDC7B. The cellular phenotype was characterized by normal development of hair cells with functional mechanotransduction complexes, followed by outer hair cell death correlated with severe and progressive hearing loss. Further work is needed to clarify the molecular function of KLHDC7B and clarify whether there is an IHC contribution to the hearing deficit.

Age-related hearing loss can not only cause social isolation and a decreased quality of life but is also the largest modifiable risk factor for dementia^68^. This emphasizes the importance of studies identifying and characterizing novel risk factors for age-related hearing loss. This study provides a strong rationale to pursue other novel candidates identified by recent ARHL focused GWAS. These studies will also provide a better understanding of the physiology of the inner ear, providing new approaches to help prevent or ameliorate hearing loss in the future.

## Methods

### Expression probe design and qPCR (quantitative polymerase chain reaction)

qPCR probes were designed using BioSearch RealTimeDesign (https://www.biosearchtech.com/support/tools/design-software/realtimedesign-software) and tested for specificity via the UCSC genome browser BLAT function. The L + S *Klhdc7b* probe was designed to a portion of the transcript overlaping the two putative isoforms, and the L *Klhdc7b* probe against a portion only present in the long isoform. For tissue collection, mice under 7 days old were decapitated; older mice were euthanized under carbon dioxide (CO_2_) with secondary decapitation. Organs were placed in RNAlater®, cochleae were frozen on dry ice. Messenger ribonucleaic acid (mRNA) was extracted and reverse-transcribed into cDNA (complementary deoxyribonucleic acid) using SuperScript® VILO™ Master Mix (Invitrogen by Life Technologies), then amplified with the SensiFAST Probe Lo-ROX (Meridian) using the 12 K Flex System (Applied Biosystems). For sequences and RNA extractions see supplemental methods.

### Animal husbandry

We have complied with all relevant ethical regulations for animal use All *Regn* mice were housed and treated according to guidelines from the Institutional Animal Care and Use Committee. All mice were crossed and bred to homozygosity with the B6.CAST-*Cdh23*^*753A>G*^ line^23^. Both male and female mice were used.

All *IMPC* mice were housed according to UK Home Office regulations. All procedures were licensed by the Home Office under the Animals (Scientific Procedures) Act 1986 and Amendment Regulations 2012 (Project Licence numbers PP7565374 and PP8324029) and approved by local institutional ethical review committees. All mice were culled using methods approved under these licences to minimize any possibility of suffering. Both male and female mice were used.

### Knockout mouse generation

For ***Klhdc7b***^***Regn***Δ/Δ^ mice*,* a targeting vector for knocking out (KO) the entire endogenous *Klhdc7b* gene was constructed using bacterial artificial chromosome (BAC) clones and VELOCIGENE® technology (see U.S. Patent No. 6,586,251)^69^. For embryonic stem (ES) cell generation and implantation, as well as sequence descriptions and primers/probes, see supplemental methods. Animals homozygous KO for the *Klhdc7b* locus were bred by crossing heterozygous animals.

The ***H-KLHDC7B-DEL1258-EM1-B6N*** mouse line (designated here as *Klhdc7b*^IMPC*-/-*^) was generated at MRC Harwell as part of the IMPC program^24^ via microinjection of CRISPR/Cas9 reagents into primed C57BL/6N oocytes which were implanted in pseudo-pregnant CD1 surrogate females. The CRISPR-induced mutation created a deletion of 1258 bp in the single exon of the *Klhdc7b* gene, within the sequence common to both long and short isoforms of *Klhdc7b*. The deletion also induces a premature stop codon creating a null allele (for further details see https://www.mousephenotype.org/data/genes/MGI:3648212). Four *IMPC*^*+/-*^ males and four *IMPC*^*+/-*^ females were imported from the MRC Mary Lyon Centre, MRC Harwell and a colony maintained at UCL. Occasionally, *IMPC*^*+/-*^ mice were outcrossed to C57BL/6 N mice with the aim to offset allelic drift. Mice were genotyped by reverse transcriptase polymerase chain reaction (rt-PCR) on cDNA made from ear biopsies to detect wildtype and mutant alleles – for primers and further details of mouse construction see Supplemental Methods.

### Histology: tissue processing and staining

#### Animal sacrifice and cochlea treatment

For *Klhdc7b*^*Regn*Δ/Δ^ mice, animals 21 days and older were sacrificed by transcardial perfusion with phosphate buffered saline (PBS) followed by 4% paraformaldehyde (PFA) in PBS. The cochleae were extracted from the temporal bone. The apex was pierced, the stapes removed and oval window pierced. Cochleae were placed 1–4 h (h) or overnight in 4% PFA followed by 3x PBS washes and stored at 4 °C until decalcification with Immunocal® (Statbio) overnight, then rinsed 3x with PBS.

For UCL *Klhdc7b*^*IMPC*^ mice, animals were sacrificed according to Schedule 1 procedures as described in the United Kingdom (Scientific Procedures) Act of 1986. For preparation of cochlea, whole inner ear was dissected (Montgomery and Cox, 2016) and fixed in 4% paraformaldehyde (PFA) diluted in 10 mM phosphate buffered saline (PBS) pH 7.4 for 2 h. Samples older than P4 were decalcified in 4% Ethylenediaminetetraacetic acid (EDTA) for 48 h.

#### Whole mount immunostaining

For immunohistochemistry of whole-mounted Organ of Corti, the sensory epithelium was dissected after decalcification. One of two protocols were used. For *Klhdc7b*^*IMPC*^ data, tissue was permeabilized with 5% tween-20 in PBS for 1 h at room temperature and blocked with 10% goat serum, 0.5% Triton-X 100 in PBS for 2 h at room temperature. Primary antibody incubation was performed overnight at 4 °C. After 3 × 15-min (min) PBS washes, secondary antibodies were incubated for 1 h at room temperature in the dark. Nuclei were visualized with 1 µM 4′,6-diamidino-2-phenylindole (DAPI), 10 nM phalloidin-Atto 647 N (Sigma-Aldrich, Gillingham, UK).

For *Klhd7cb*^*Regn*^ data, dissected samples were washed 3x in PBS, incubated with blocking solution for 1 h at room temperature, incubated overnight with primary antibodies in blocking solution at 4 °C or room temperature, then washed 3x with PBS. Secondary antibodies and cell stains were diluted in blocking buffer and samples were incubated for 1 h at room temperature, rinsed 3x with PBS, then placed on slides and covered with Prolong gold and a coverslip. Primary antibodies used were from the following suppliers anti-Myo7A, Proteus #25-6790; anti-Sox2, BD Pharmingen #AB_1645334; anti-Ctbp2, BD Transduction Labs #612044; anti-GluA2, Millipore #MAB397, anti-KLHDC7B is a custom antibody generated against the entire putative short isoform of the human protein. A full list of antibodies and dilutions is given in the Supplemental Methods. For Fig. 1E, F, the synaptic staining protocol was used, see Supplemental Methods: Whole mount and synaptic staining and quantification.

#### Paraffin embedding, slicing and immunostaining

For paraffin embedding, cochleae were placed in cassettes, dehydrated and immersed in a vacuum chamber containing warm paraffin wax using a tissue processor (Leica). The protocol was 70% ethanol for 10 min, 95% ethanol for 15 min, 95% ethanol for 12 min, 100% ethanol for 12 min × 3, xylene × 12 min, xylene × 15 min × 2, and paraffin × 30 min, then 35 min. Cochleae were embedded in paraffin, sliced using a microtome at six-micron thickness and dried on a heat block. For immunostaining, slides were baked at 60 °C for 1 h, washed in xylene 2x for 3 min, and rehydrated in 100% ethanol 2 × 3 min, 95% ethanol 2 × 3 min, 70% ethanol for 3 min, 50% ethanol for 3 min, distilled water for 5 min, and 3 × 2 min PBS. For antigen retrieval, Co-detection target retrieval reagent (ACD Bio) was heated in a vegetable steamer (Oster), and slides were placed in hot target retrieval reagent for 20 min in the steamer, cooled for 10 min, and washed 2x with PBS plus 0.5% Tween-20.

A hydrophobic barrier was drawn around the sections and slides were incubated at room temperature in blocking solution (2% weight by volume, w/v, bovine serum albumin, 5% normal donkey serum, 0.01% triton-x 100 in PBS), in a humidified chamber for 30 min. Primary antibodies were diluted in blocking solution, added to the slides and incubated at 4 °C overnight followed by 3x PBS washes, then incubated with secondary antibodies and cell stains diluted in blocking solution for 1 h. Slides were washed 3x in PBS, then mounted as above.

#### Cryoembedding and staining

For cryoembedding and sectioning, decalcified cochleae were dehydrated in 15% and 30% sucrose and embedded in optimal cutting temperature media in liquid nitrogen. Cryoembedded samples were sectioned at 10 µm using a OTF6000 Cryostat (Bright Instruments, UK). Mid-modiolar sections was collected for immunostaining. Cryosections were rehydrated in PBS for 10 min and incubated in 1% sodium dodecyl sulfate (SDS) for 5 min to retrieve antigen at room temperature. After 3 × 10 min washes in PBS, sections were permeabilized and blocked in blocking buffer (10% goat serum, 0.1% Triton X-100 in PBS) for 1 h at room temperature. Sections were incubated with primary antibodies overnight at 4 °C, followed by 3 × 10 min PBS-T (0.1% Triton X-100 in PBS) washes. Sections were incubated for 2 h at room temperature in secondary antibodies and DAPI were diluted in blocking buffer. After 3x washes with PBS-T, sections were mounted in Fluoromount-G® (SouthernBiotech).

#### RNAscope with antibody co-detection

For RNA scope in paraffin embedded sections, the ACD Bio protocol for paraffin embedded slides was followed using the suggested standard incubation times for antigen retrieval. Antibody (anti-MYO7A, Proteus, 25-6790) was diluted at a concentration of 1:200 in Co-detection antibody diluent and incubated overnight at 4  °C in a humidified chamber. Opal 520 and 570 dyes were used at 1:1500. For details on reagents, see Supplemental Methods.

#### LacZ staining of knockout mice

Mice were anesthetized with ketamine/xylazine and transcardially perfused with PBS followed by 2% paraformaldehyde. Tissues were dissected, post-fixed 30 min at room temperature, washed in PBS for 30 min and stained in beta galactosidase (LacZ) solution overnight at 4 °C. After staining, tissues were washed in cold PBS for 15 min and postfixed in 4% formaldehyde at 4 °C overnight with mixing. Tissues were incubated in 50% glycerol then 70% glycerol for one day each at room temperature and stored in 70% glycerol. Cochlea were removed, decalcified overnight with Immunocal®, rinsed and dissected, then photographed using an Axioscan slide scanner (Zeiss).

#### *Klhdc7b*^*IMPC*^ hair cell counts

Hair cell counts were performed on maximum intensity projections of confocal Z-stacks collected at 1.0 µm intervals (63x oil, 1.4 N.A. objective) from a minimum of 3 mice for each genotype and age. Data were collected from mid-basal, mid-middle and mid-apical cochlear turns correlated to the mouse tonotopic frequency-place map^70^. Data were quantified in 250 µm lengths and individual hair cells were counted on 3 separate occasions to ensure consistency.

### Confocal and Airyscan microscopy

Samples were imaged on a confocal microscope; either Zeiss LSM 780 or Zeiss LSM 880 using 10x (0.45 N.A.), 20x (0.8 N.A.), and 63x oil (1.4 N.A.), and 100x oil (1.4 N.A.) objectives and Zen 3.0 SR software (Black edition). For Fig. 1E–H, Zeiss LSM 980 with Airyscan acquisition and processing were used, using Zen Blue software. Z-stacks were taken with the appropriate size for the objective and numerical aperture. Image processing and analysis were performed with Fiji. For display, any adjustments were made equally to images from all 3 genotypes. All immunostaining experiments used a minimum of 3 mice per genotype and age range, representative images are shown in the figures.

### Scanning Electron Microscopy (SEM) and Transmission Electron Microscopy (TEM)

For SEM of *Klhdc7b*^*Regn*^ mice*,* cochleae were removed and fixed overnight in 3% formaldehyde, 3% glutaraldehyde in 0.1 M sodium cacodylate, pH 7.4 (Electron Microscopy Sciences, Hatfield, PA) with 2 mM CaCl_2_. Samples were washed with 0.1 M sodium cacodylate, decalcified for 24 h in Immunocal, washed 3× with PBS and dissected to three flat turns. Bone, strial ligament and tectorial membrane were removed and cochlear turns were placed into porous baskets and dehydrated in ethanol (5%, 10%, 20%, 40%, 60%, 80% and 100%), critical-point dried using a manual CPD (BAL-TEC 030) in liquid CO_2_, sputter coated with 5 nm Platinum and imaged with a field-emission scanning electron microscope (Zeiss Sigma VP).

For SEM of *Klhdc7b*^*IMPC*^ mice, preparation of the organ of Corti was performed as in previously described^71^. Cochleae were fixed (2 h, 2.5% glutaraldehyde, 2% PFA 0.1 M cacodylate buffer, 3 mM CaCl_2_, room temperature), decalcified (48 h, 4% EDTA, 4 °C) and the organ of Corti dissected, post-fixed in OsO_4_ and processed through the thiocarbohydrazide-Os-repeated procedure. Processed samples were dehydrated in a graded ethanol series, critical point-dried and sputter coated with platinum. Samples were examined in a JEOL 6700 F SEM operating at 5 kV by secondary electron detection. Imaging was carried out using SEM Supporter software (System In Frontier, Japan).

For TEM, samples were prepared as described in Bullen, et al.^71^. Samples were fixed and decalcified before post-fixation in OsO_4_, then dehydrated in 30% and 50% ethanol for 15 min each at room temperature. En-bloc staining was performed using 2% uranyl acetate in 70% ethanol at 4 °C overnight. Cochlea were processed in 85%, 95%, 100% ethanol and propylene oxide for 15 min each, followed by incubation in 25% over-day, 50% overnight and 75% resin over-day. Cochleae were then embedded in 100% Agar100 Epon resin (Agar Scientific, UK) and let cure for 24 h at 60 °C. Resin blocks were trimmed and cut in at 110 nm using Ultramicrotome (Reichert). 100 nm sections were post-stained with uranyl acetate and lead citrate, before examination on a JEOL 1400Flash transmission electron microscope at 120 kV with a Gatan RIO16 camera.

### 3D microscopy

Cochleae were decalcified and bone carefully cut around the spiral for antibody penetration. Cochleae were stained with anti-MYO7A and Sytox Deep red, a nuclear stain, embedded in agarose, and dehydrated using a methanol series, cleared in ethyl cinnamate (ECi) and imaged using an Ultramicroscope Blaze (Miltenyi). Image files were converted to Imaris format, and hair cells were virtually dissected^30^. Inner and outer hair cell masks were created, and data within the masks were exported to Python. Hair cells were extracted using a custom cell segmentation algorithm. A custom processing pipeline was developed to unwrap the cochlea and normalize it to the tonotopic map. For details, see Supplemental Methods.

### Behavioral assays

Mice were tested on open field, rotarod, pole test, and a Y-maze on different days. For details, see Supplemental Methods.

### Auditory brainstem response (ABR)

For *Klhdc7b*^*Regn*^ mice, ABRs were performed in a soundproof booth (IAC Acoustics, IL) at three pure-tone frequencies (8 kHz, 16 kHz, and 32 kHz) using the TDT RZ6 recording system (Tucker-Davis Technologies, FL), with 512 presentations of each stimulus averaged at each sound-pressure-level. Stimuli were presented for 2.5 milliseconds (ms) with a 0.2 ms cos^2^ rise-decay with a Hanning window applied at a rate of 21 presentations per second. Each frequency was played starting at 90 decibels (dB SPL), then 10 dB decrements to 50 dB and 5 dB decrements from 50 dB to 15 dB. ABR waveforms were bandpass filtered between 300 Hz and 3 kHz with a 60 Hz notch filter. Recordings were processed in Matlab. Traces were smoothed with a moving median filter with a kernel of 50 time points (each 10 ms recordings contained 244 time points) to remove occasionally observed slow wave noise. Thresholds were run through an algorithm adapted from the Liberman lab^72^ and validated manually. Genotypes were interleaved during recordings. See Supplemental Methods for details.

For *Klhdc7b*^*IMPC*^ mice, ABRs were performed at MRC Harwell using a click stimulus in addition to frequency-specific tone-burst stimuli. ABR responses were collected, amplified, and averaged using TDT System 3 (Tucker Davies Technology) in conjunction with BioSig RZ (v5.7.1) software. The TDT system click ABR stimuli comprised clicks of 0.1 ms broadband noise spanning approximately 2–48 kHz, presented at a rate of 21.1 s^−1^ with alternating polarity. Tone-burst stimuli were of 7 ms duration, inclusive of 1 ms rise/fall gating using a Cos^2^ filter, presented at a rate of 42.5 s^−1^ and were measured at 8, 16 and 32 kHz. All stimuli were presented free-field with the speaker facing toward the right ear, starting at 90 dB SPL and decreasing in 5 dB steps. Thresholds were manually determined as the lowest dB SPL that produced a reproducible ABR trace pattern. All ABR waveform traces were re-scored by a second operator blinded to genotype. For anesthesia and recovery, see Supplemental Methods.

### Distortion product otoacoustic emissions

For *Klhdc7b*^*Regn*^ mice, DPOAE measurements were taken using the same system as ABRs. Two speakers were used to present two primary tones (f1:f2 = 1.2) equally spaced around three center frequencies measured for ABR (8 kHz, 16 kHz, and 32 kHz), presented 100 times and averaged. The speakers were used in a closed-field configuration, with tubes connecting the speaker to a sensitive microphone (DPM1, Tucker-Davis Technologies, FL) placed inside the ear canal angled toward the eardrum. Stimuli were calibrated in the ear within 2 dB of the target level. The distortion product was detected at the expected cubic distortion frequency (2f1 − f2) and called as positive if the signal was above the noise floor. The noise floor was calculated as the average of five points in the Fourier transform above and below the cubic distortion tone frequency. Genotypes were interleaved during recordings.

For *Klhdc7b*^*IMPC*^ mice, DPOAE tests were performed at MRC Harwell using frequency-specific tone-burst stimuli at 2 kHz intervals, between 6 and 32 kHz, using the same system as ABRs. An ER10B+ low noise probe microphone (Etymotic Research) was used to measure the DPOAE near the tympanic membrane. Tone stimuli were presented via separate MF1 (Tucker Davis Technology) speakers, with f1 and f2 at a ratio of f2/f1 = 1.2 (L1 = 65 dB SPL, L2 = 55 dB SPL), centered around the probing frequencies. In-ear calibration was performed before each test. The f1 and f2 tones were presented continuously and a fast-Fourier transform was performed on the averaged response of 356 epochs (each approximately 21 ms). The level of the 2f1 − f2 DPOAE response was recorded and the noise floor calculated by averaging the four frequency bins either side of the 2f1 − f2 frequency.

### Cochlear explant cultures

Cochlear explant cultures were performed with mice at postnatal days 0–5 and placed on a collagen bubble in a 35 mm dish with a punchout and glass coverslip glued to the bottom (Matsunami, #D35-14-0-U). For collagen and media composition see supplemental methods.

The mice were decapitated and cochlea removed and placed in Leibovitz/L15 media. The bone was opened, the organ of Corti removed and stria vascularis separated. PBS was removed from the culture plate and replaced with cochlear explant media. The organ of Corti was placed on the collagen bubble and most of the media removed to allow the explant to adhere to the collagen. Plates were placed in a humidified incubator at 37 °C with 5% CO_2_. After dissections were finished, 600 μl of media was added.

### Gentamycin-Texas red assay

Explant cultures were kept in culture for 2–3 days and treated with gentamycin-Texas Red (GtTR, 5 μg/ml) or Texas red (TR, 12.5 μg/ml) diluted in cell culture media for 20 min in the cell culture incubator, rinsed with media, PBS, then fixed for 15 min in 4% PFA, rinsed 3x with PBS and stored at 4 °C until immunostained as whole mounts above.

### Statistics and reproducibility

Where applicable, student’s T-test, one or two-way ANOVA (Analysis of Variance) as indicated was used to analyze data for significance. All datasets are tested for normality and if failed, non-parametric tests used: Mann–Whitney and Kruskal-Wallis tests. Repeated measures or mixed models were used if data points were missing. If a significant main effect of any variable was observed, Tukey post-hoc tests were performed. Statistics were calculated using GraphPad Prism. For raw data, descriptive statistics, details of tests used, n by sex for mice for each figure, exclusion criteria if applicable, please see statistical summary file in Supplementary data 1.

### Variant effect predictions and protein modeling

Ensembl Variant Effect Predictor^73^ was used to generate protein predictions for the effect of the rs36062310 missense variant on KLHDC7B. Alphafold (https://alphafold.ebi.ac.uk/) results for both human isoforms are available with their unique Uniprot IDs (Long variant: A0A3B3ISF6; Short variant: Q96G42). The *.PDB files were downloaded for both human KLHDC7B isoforms and utilized for comparative in-silico protein structure modeling using RCSB Protein Data Bank (RCSB PDB)^74^. and Missense3D^67^.

### Reporting summary

Further information on research design is available in the Nature Portfolio Reporting Summary linked to this article.

## Supplementary information

Supplementary Information

Description of Additional Supplementary Files

Supplementary Data 1

Reporting Summary

## Data Availability

The datasets and materials used and/or analyzed during the current study are available on reasonable request to the authors, subject to an MTA where necessary. The source data can be found within Supplementary Data 1. The *Klhdc7b*^*IMPC-/-*^ mice are available from the IMPC. Regeneron materials described in this manuscript may be available to qualified, academic, non-commercial researchers upon request through our portal (https://regeneron.envisionpharma.com/ienv_research/visiontracker/portal/login.xhtml?pgm=ISR&windowId=4d6). Regeneron does not share clinical molecules. Regeneron does share alternative molecules that behave in a similar manner. For any questions about how Regeneron shares materials please connect with Regeneron using the preclinical collaborations email address (preclinical.collaborations@regeneron.com).

## References

[1] Livingston, G. et al. Dementia prevention, intervention, and care. *Lancet* **390**, 2673–2734 (2017). 28735855 10.1016/S0140-6736(17)31363-6

[2] Livingston, G. et al. Dementia prevention, intervention, and care: 2020 report of the Lancet Commission. *Lancet* **396**, 413–446 (2020). 32738937 10.1016/S0140-6736(20)30367-6 PMC7392084

[3] Livingston, G. et al. Dementia prevention, intervention, and care: 2024 report of the Lancet standing Commission. *Lancet* **404**, 572–628 (2024). 39096926 10.1016/S0140-6736(24)01296-0

[4] Collaborators, G. D. F. Estimation of the global prevalence of dementia in 2019 and forecasted prevalence in 2050: an analysis for the Global Burden of Disease Study 2019. *Lancet Public Health* **7**, e105–e125 (2022). 34998485 10.1016/S2468-2667(21)00249-8 PMC8810394

[5] *World Health Organization: Fact Sheet on Deafness and Hearing Loss* https://www.who.int/news-room/fact-sheets/detail/deafness-and-hearing-loss (2025).

[6] Bowl, M. R. & Dawson, S. J. Age-related hearing loss. *Cold Spring Harb. Perspect. Med.* **9**, 10.1101/cshperspect.a033217 (2019). 10.1101/cshperspect.a033217 PMC6671929 30291149

[7] Friedman, R. A. et al. GRM7 variants confer susceptibility to age-related hearing impairment. *Hum. Mol. Genet.* **18**, 785–796 (2009). 19047183 10.1093/hmg/ddn402 PMC2638831

[8] Van Laer, L. et al. A genome-wide association study for age-related hearing impairment in the Saami. *Eur. J. Hum. Genet.* **18**, 685–693 (2010). 20068591 10.1038/ejhg.2009.234 PMC2987344

[9] Wolber, L. E. et al. Salt-inducible kinase 3, SIK3, is a new gene associated with hearing. *Hum. Mol. Genet.* **23**, 6407–6418 (2014). 25060954 10.1093/hmg/ddu346 PMC4222365

[10] Fransen, E. et al. Genome-wide association analysis demonstrates the highly polygenic character of age-related hearing impairment. *Eur. J. Hum. Genet.* **23**, 110–115 (2015). 24939585 10.1038/ejhg.2014.56 PMC4266741

[11] Vuckovic, D. et al. Genome-wide association analysis on normal hearing function identifies PCDH20 and SLC28A3 as candidates for hearing function and loss. *Hum. Mol. Genet.* **24**, 5655–5664 (2015). 26188009 10.1093/hmg/ddv279 PMC4572074

[12] Hoffmann, T. J. et al. A large genome-wide association study of age-related hearing impairment using electronic health records. *PLoS Genet.* **12**, e1006371 (2016). 27764096 10.1371/journal.pgen.1006371 PMC5072625

[13] Nolan, L. S. et al. Estrogen-related receptor gamma and hearing function: evidence of a role in humans and mice. *Neurobiol. Aging* **34**, 2077.e2071–2077.e2079 (2013). 10.1016/j.neurobiolaging.2013.02.009 PMC4330334 23540940

[14] Kalra, G. et al. Biological insights from multi-omic analysis of 31 genomic risk loci for adult hearing difficulty. *PLoS Genet.* **16**, e1009025 (2020). 32986727 10.1371/journal.pgen.1009025 PMC7544108

[15] Trpchevska, N. et al. Genome-wide association meta-analysis identifies 48 risk variants and highlights the role of the stria vascularis in hearing loss. *Am. J. Hum. Genet.* **109**, 1077–1091 (2022). 35580588 10.1016/j.ajhg.2022.04.010 PMC9247887

[16] Wells, H. R. R. et al. GWAS identifies 44 Independent Associated Genomic Loci for self-reported adult hearing difficulty in UK Biobank. *Am. J. Hum. Genet.* **105**, 788–802 (2019). 31564434 10.1016/j.ajhg.2019.09.008 PMC6817556

[17] Cornejo-Sanchez, D. M. et al. Rare-variant association analysis reveals known and new age-related hearing loss genes. *Eur. J. Hum. Genet* **31**, 638–647 (2023). 36788145 10.1038/s41431-023-01302-2 PMC10250305

[18] Praveen, K. et al. Population-scale analysis of common and rare genetic variation associated with hearing loss in adults. *Commun. Biol.* **5**, 540 (2022). 35661827 10.1038/s42003-022-03408-7 PMC9166757

[19] Boucher, S. et al. Ultrarare heterozygous pathogenic variants of genes causing dominant forms of early onset deafness. *PNAS* **117**, 31278–31289 (2020). 33229591 10.1073/pnas.2010782117 PMC7733833

[20] Adams, J., Kelso, R. & Cooley, L. The kelch repeat superfamily of proteins: propellers of cell function. *Trends Cell Biol.* **10**, 17–24 (2000). 10603472 10.1016/s0962-8924(99)01673-6

[21] Gupta, V. A. & Beggs, A. H. Kelch proteins: emerging roles in skeletal muscle development and diseases. *Skelet. Muscle* **4**, 1–12 (2014). 24959344 10.1186/2044-5040-4-11 PMC4067060

[22] Martin-Pardillos, A. & Cajal, S. R. Y. Characterization of Kelch domain-containing protein 7B in breast tumours and breast cancer cell lines. *Oncol. Lett.* **18**, 2853–2860 (2019). 31452764 10.3892/ol.2019.10672 PMC6704290

[23] Johnson, K. R., Erway, L. C., Cook, S. A., Willott, J. F. & Zheng, J. A major gene affecting age-related hearing loss in C57BL/6J mice. *Hearing Res.* **114**, 83–92 (1997). 10.1016/s0378-5955(97)00155-x 9447922

[24] Groza, T. et al. The International Mouse Phenotyping Consortium: comprehensive knockout phenotyping underpinning the study of human disease. *Nucleic Acids Res.* **51**, D1038–D1045 (2023). 36305825 10.1093/nar/gkac972 PMC9825559

[25] Bowl, M. R. et al. A large scale hearing loss screen reveals an extensive unexplored genetic landscape for auditory dysfunction. *Nat. Commun.* **8**, 886 (2017). 29026089 10.1038/s41467-017-00595-4 PMC5638796

[26] Elrick, H. et al. Impact of essential genes on the success of genome editing experiments generating 3313 new genetically engineered mouse lines. *Sci. Rep.* **14**, 22626 (2024). 10.1038/s41598-024-72418-8 PMC11443006 39349521

[27] Wagner, E. L. & Shin, J. B. Mechanisms of hair cell damage and repair. *Trends Neurosci.* **42**, 414–424 (2019). 30992136 10.1016/j.tins.2019.03.006 PMC6556399

[28] Monzack, E. L. & Cunningham, L. L. Lead roles for supporting actors: critical functions of inner ear supporting cells. *Hear Res.* **303**, 20–29 (2013). 23347917 10.1016/j.heares.2013.01.008 PMC3648608

[29] Monzack, E. L., May, L. A., Roy, S., Gale, J. E. & Cunningham, L. L. Live imaging the phagocytic activity of inner ear supporting cells in response to hair cell death. *Cell Death Differ.* **22**, 1995–2005 (2015). 25929858 10.1038/cdd.2015.48 PMC4816108

[30] Hutson, K. A., Pulver, S. H., Ariel, P., Naso, C. & Fitzpatrick, D. C. Light sheet microscopy of the gerbil cochlea. *J. Comp. Neurol.* **529**, 757–785 (2021). 32632959 10.1002/cne.24977 PMC7775904

[31] Fang, Q. et al. The 133-kDa N-terminal domain enables myosin 15 to maintain mechanotransducing stereocilia and is essential for hearing. *Elife* **4**, 10.7554/eLife.08627 (2015). 10.7554/eLife.08627 PMC4592939 26302205

[32] Belyantseva, I. A. et al. Myosin-XVa is required for tip localization of whirlin and differential elongation of hair-cell stereocilia. *Nat. Cell Biol.* **7**, 148–156 (2005). 15654330 10.1038/ncb1219

[33] Mburu, P. et al. Defects in whirlin, a PDZ domain molecule involved in stereocilia elongation, cause deafness in the whirler mouse and families with DFNB31. *Nat. Genet.* **34**, 421–428 (2003). 12833159 10.1038/ng1208

[34] Manor, U. et al. Regulation of stereocilia length by myosin XVa and whirlin depends on the actin-regulatory protein Eps8. *Curr. Biol.* **21**, 167–172 (2011). 21236676 10.1016/j.cub.2010.12.046 PMC3040242

[35] Zampini, V. et al. Eps8 regulates hair bundle length and functional maturation of mammalian auditory hair cells. *PLoS Biol.* **9**, e1001048 (2011). 21526224 10.1371/journal.pbio.1001048 PMC3079587

[36] Marcovich, I. et al. Optimized AAV vectors for TMC1 gene therapy in a humanized mouse model of DFNB7/11. *Biomolecules* **12**, 10.3390/biom12070914 (2022). 10.3390/biom12070914 PMC9313133 35883470

[37] Alharazneh, A. et al. Functional hair cell mechanotransducer channels are required for aminoglycoside ototoxicity. *PLoS ONE* **6**, e22347 (2011). 21818312 10.1371/journal.pone.0022347 PMC3144223

[38] Marcotti, W., van Netten, S. M. & Kros, C. J. The aminoglycoside antibiotic dihydrostreptomycin rapidly enters mouse outer hair cells through the mechano-electrical transducer channels. *J. Physiol.* **567**, 505–521 (2005). 15994187 10.1113/jphysiol.2005.085951 PMC1474200

[39] Kawashima, Y. et al. Mechanotransduction in mouse inner ear hair cells requires transmembrane channel-like genes. *J. Clin. Investig.* **121**, 4796–4809 (2011). 22105175 10.1172/JCI60405 PMC3223072

[40] Makabe, A. et al. Systemic fluorescent gentamicin enters neonatal mouse hair cells predominantly through sensory mechanoelectrical transduction channels. *J. Assoc. Res Otolaryngol.* **21**, 137–149 (2020). 32152768 10.1007/s10162-020-00746-3 PMC7270392

[41] Becker, L. et al. The presynaptic ribbon maintains vesicle populations at the hair cell afferent fiber synapse. *Elife* **7**, 10.7554/eLife.30241 (2018). 10.7554/eLife.30241 PMC5794257 29328021

[42] Wu, P. Z., O’Malley, J. T., de Gruttola, V. & Liberman, M. C. Age-related hearing loss is dominated by damage to inner ear sensory cells, not the cellular battery that powers them. *J. Neurosci.* **40**, 6357–6366 (2020). 32690619 10.1523/JNEUROSCI.0937-20.2020 PMC7424870

[43] Walls, W., Azaiez, H. & Smith, R. *Hereditary Hearing Loss Homepage*, https://hereditaryhearingloss.org.

[44] Ashmore, J. Cochlear outer hair cell motility. *Physiol. Rev.* **88**, 173–210 (2008). 18195086 10.1152/physrev.00044.2006

[45] Chen, G. D. & Fechter, L. D. The relationship between noise-induced hearing loss and hair cell loss in rats. *Hear Res.* **177**, 81–90 (2003). 12618320 10.1016/s0378-5955(02)00802-x

[46] Maraslioglu-Sperber, A., Blanc, F., Heller, S. & Benkafadar, N. Hyperosmotic sisomicin infusion: a mouse model for hearing loss. *Sci. Rep.* **14**, 15903 (2024). 38987330 10.1038/s41598-024-66635-4 PMC11237112

[47] Furness, D. N. Molecular basis of hair cell loss. *Cell Tissue Res.* **361**, 387–399 (2015). 25676005 10.1007/s00441-015-2113-z

[48] Shnerson, A., Devigne, C. & Pujol, R. Age-related changes in the C57BL/6J mouse cochlea. II. Ultrastructural findings. *Brain Res.* **254**, 77–88 (1981). 7272774 10.1016/0165-3806(81)90060-2

[49] Shnerson, A. & Pujol, R. Age-related changes in the C57BL/6J mouse cochlea. I. Physiological findings. *Brain Res.* **254**, 65–75 (1981). 7272773 10.1016/0165-3806(81)90059-6

[50] Wu, X., Gao, J., Guo, Y. & Zuo, J. Hearing threshold elevation precedes hair-cell loss in prestin knockout mice. *Brain Res. Mol. Brain Res.* **126**, 30–37 (2004). 15207913 10.1016/j.molbrainres.2004.03.020

[51] Tong, B. et al. Oncomodulin, an EF-Hand Ca2+ buffer, is critical for maintaining cochlear function in mice. *J. Neurosci.* **36**, 1631–1635 (2016). 26843644 10.1523/JNEUROSCI.3311-15.2016 PMC4737773

[52] Murtha, K. E. et al. Oncomodulin (OCM) uniquely regulates calcium signaling in neonatal cochlear outer hair cells. *Cell Calcium* **105**, 102613 (2022). 35797824 10.1016/j.ceca.2022.102613 PMC9297295

[53] Zhou, H. et al. Disruption of Atg7-dependent autophagy causes electromotility disturbances, outer hair cell loss, and deafness in mice. *Cell Death Dis.* **11**, 913 (2020). 33099575 10.1038/s41419-020-03110-8 PMC7585579

[54] Herranen, A. et al. Deficiency of the ER-stress-regulator MANF triggers progressive outer hair cell death and hearing loss. *Cell Death Dis.* **11**, 100 (2020). 32029702 10.1038/s41419-020-2286-6 PMC7005028

[55] Asamura, K. et al. Type IX collagen is crucial for normal hearing. *Neuroscience* **132**, 493–500 (2005). 15802199 10.1016/j.neuroscience.2005.01.013

[56] Jeng, J. Y. et al. MET currents and otoacoustic emissions from mice with a detached tectorial membrane indicate the extracellular matrix regulates Ca(2+) near stereocilia. *J. Physiol.* **599**, 2015–2036 (2021). 33559882 10.1113/JP280905 PMC7612128

[57] Kall, L., Krogh, A. & Sonnhammer, E. L. A combined transmembrane topology and signal peptide prediction method. *J. Mol. Biol.* **338**, 1027–1036 (2004). 15111065 10.1016/j.jmb.2004.03.016

[58] Kall, L., Krogh, A. & Sonnhammer, E. L. Advantages of combined transmembrane topology and signal peptide prediction-the Phobius web server. *Nucleic Acids Res.* **35**, W429–W432 (2007). 17483518 10.1093/nar/gkm256 PMC1933244

[59] Triffo, W. J. et al. 3D ultrastructure of the cochlear outer hair cell lateral wall revealed by electron tomography. *Front. Cell Neurosci.* **13**, 560 (2019). 31920560 10.3389/fncel.2019.00560 PMC6933316

[60] Newton, S. et al. Neuroplastin genetically interacts with Cadherin 23 and the encoded isoform Np55 is sufficient for cochlear hair cell function and hearing. *PLoS Genet.* **18**, e1009937 (2022). 35100259 10.1371/journal.pgen.1009937 PMC8830789

[61] Newton, S. et al. Absence of Embigin accelerates hearing loss and causes sub-viability, brain and heart defects in C57BL/6N mice due to interaction with Cdh23(ahl). *iScience* **26**, 108056 (2023). 37854703 10.1016/j.isci.2023.108056 PMC10579432

[62] Newton, S., Aguilar, C. & Bowl, M. R. C57BL/6-derived mice and the Cdh23(ahl) allele - Background matters. *Hear Res.* **462**, 109278 (2025). 40305983 10.1016/j.heares.2025.109278

[63] Wu, P., Wen, W., O’Malley, J. T. & Liberman, M. C. Assessing fractional hair cell survival in archival Huma. *Laryngoscope* **130**, 487–495 (2020). 30963586 10.1002/lary.27991 PMC6783317

[64] Milon, B. et al. A cell-type-specific atlas of the inner ear transcriptional response to acoustic trauma. *Cell Rep.* **36**, 109758 (2021). 34592158 10.1016/j.celrep.2021.109758 PMC8709734

[65] Ivarsdottir, E. V. et al. The genetic architecture of age-related hearing impairment revealed by genome-wide association analysis. *Commun. Biol.* **4**, 706 (2021). 34108613 10.1038/s42003-021-02224-9 PMC8190123

[66] De Angelis, F. et al. Sex differences in the polygenic architecture of hearing problems in adults. *Genome Med.* **15**, 36 (2023). 37165447 10.1186/s13073-023-01186-3 PMC10173489

[67] Ittisoponpisan, S. et al. Can predicted protein 3D structures provide reliable insights into whether missense variants are disease associated? *J. Mol. Biol.* **431**, 2197–2212 (2019). 30995449 10.1016/j.jmb.2019.04.009 PMC6544567

[68] Griffiths, T. D. et al. How can hearing loss cause dementia? *Neuron* **108**, 401–412 (2020). 32871106 10.1016/j.neuron.2020.08.003 PMC7664986

[69] Valenzuela, D. M. et al. High-throughput engineering of the mouse genome coupled with high-resolution expression analysis. *Nat. Biotechnol.* **21**, 652–659 (2003). 12730667 10.1038/nbt822

[70] Muller, M., Von Hunerbein, K., Hoidis, S. & Smolders, J. W. A physiological place-frequency map of the cochlea in the CBA/J mouse. *Hear Res.* **202**, 63–73 (2005). 15811700 10.1016/j.heares.2004.08.011

[71] Bullen, A. et al. Ultrastructural defects in stereocilia and tectorial membrane in aging mouse and human cochleae. *J. Neurosci. Res.* **98**, 1745–1763 (2020). 31762086 10.1002/jnr.24556

[72] Suthakar, K. & Liberman, M. C. A simple algorithm for objective threshold determination of auditory brainstem responses. *Hear Res.* **381**, 107782 (2019). 31437652 10.1016/j.heares.2019.107782 PMC6726521

[73] McLaren, W. et al. The ensembl variant effect predictor. *Genome Biol.* **17**, 122 (2016). 27268795 10.1186/s13059-016-0974-4 PMC4893825

[74] Berman, H. et al. The protein Data Bank. *Nucleic Acids Res.* **28**, 235–242 (2000). 10592235 10.1093/nar/28.1.235 PMC102472

