# Supplementary material for: Cross-species validation of a human age-related hearing loss candidate KLHDC7B as essential for mammalian hearing
Source: Commun Biol. 2025 Dec 17;9:84. doi: 10.1038/s42003-025-09349-1 (PMC12820229; doi:10.1038/s42003-025-09349-1)
Supplement: Supplementary file 1 — Supplementary Information [file 42003_2025_9349_MOESM1_ESM.pdf]

Supplementary Figures

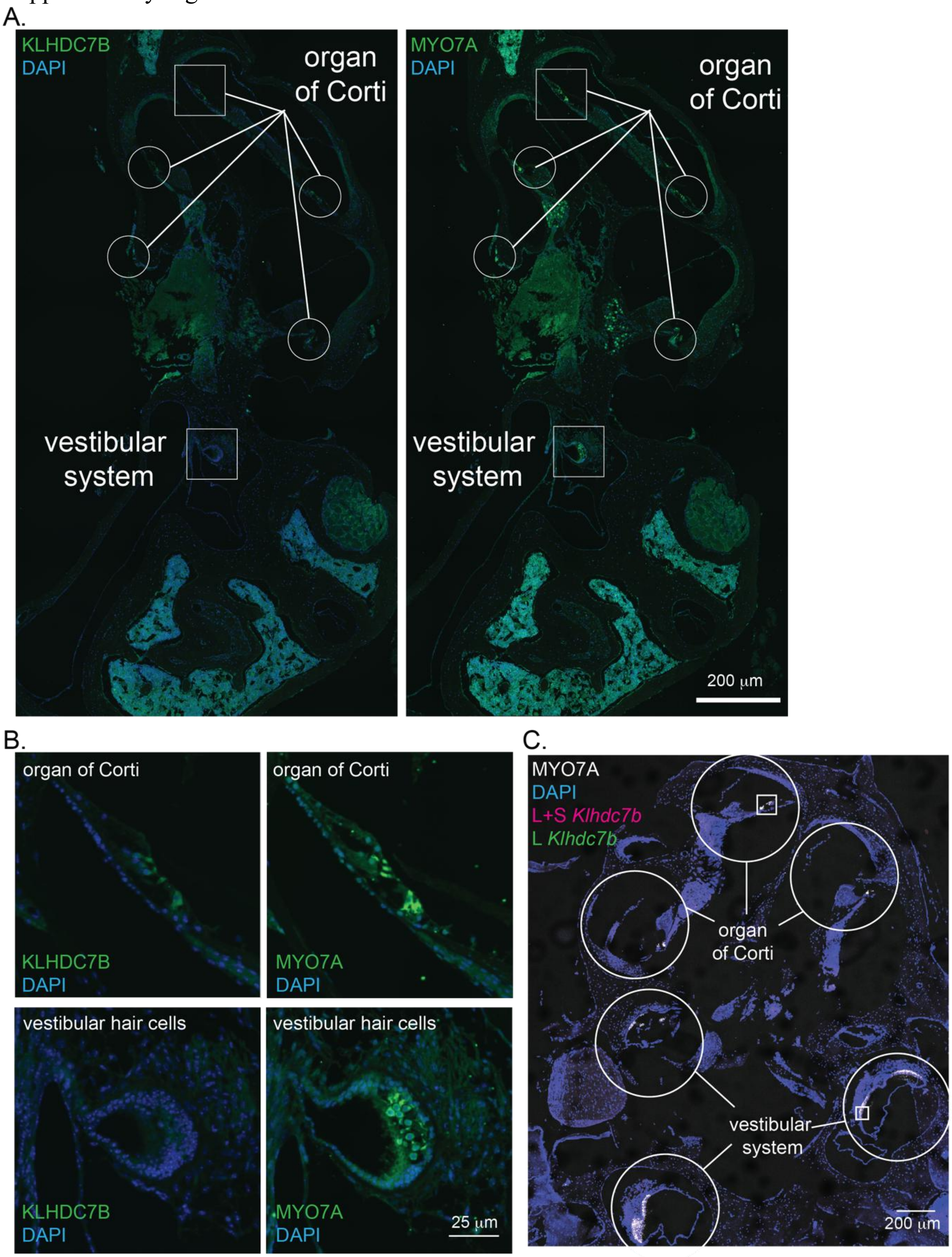

Supplementary Figure 1. Immunostaining and RNAScope in whole cochlea. A. An adult mouse with serial sections immunostained against KLHDC7B (left) and MYO7A (right) to determine hair cell expression with immunofluorescence. Boxes in **A** highlight one turn of the organ of Corti and the crista ampullaris, with closer views of each presented in **B. C.** RNAScope of the whole cochlea presented in main figure 1C, with the circles indicating hair cell locations in the organ of Corti or vestibular system, and boxes highlighting organ of Corti and vestibular areas shown in closeup in figure 1C.

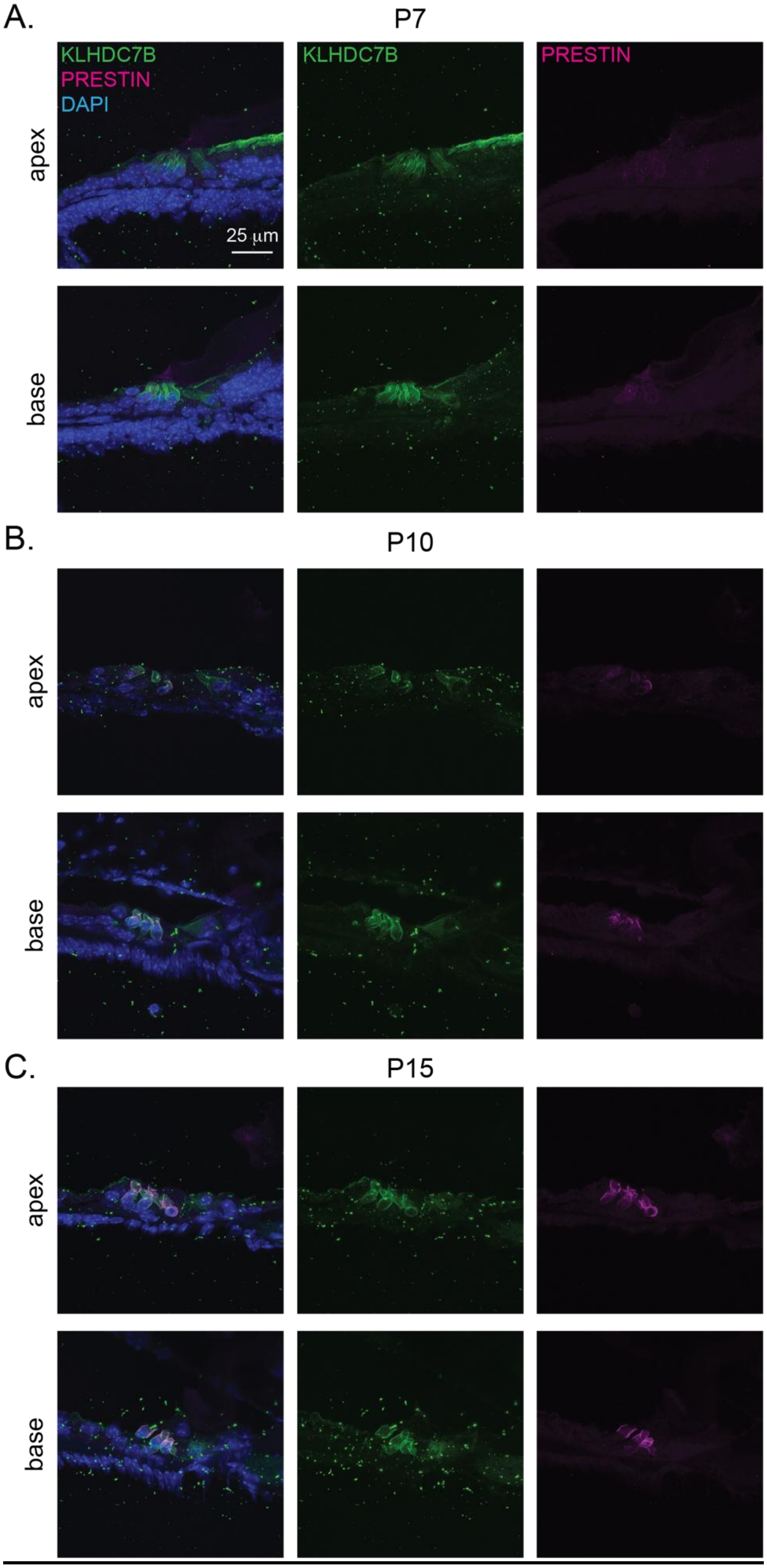
A.
P7
KLHDC7B
PRESTIN
DAPI
KLHDC7B
PRESTIN
apex
25 μm
base
B.
P10
apex
base
C.
P15
apex
base

11

Supplementary Figure 2. Developmental expression of KLHDC7B and prestin. Cochleae of an animal at **A.** postnatal day 7 (P7), **B.** postnatal day 10 (P10), and **C.** postnatal day 15 (P15), each with an image from the apex (top row) and base (bottom row) of the cochlea imaged at 63x. Leftmost column is an overlay of three channels, middle column shows KLHDC7B, and right column shows prestin.

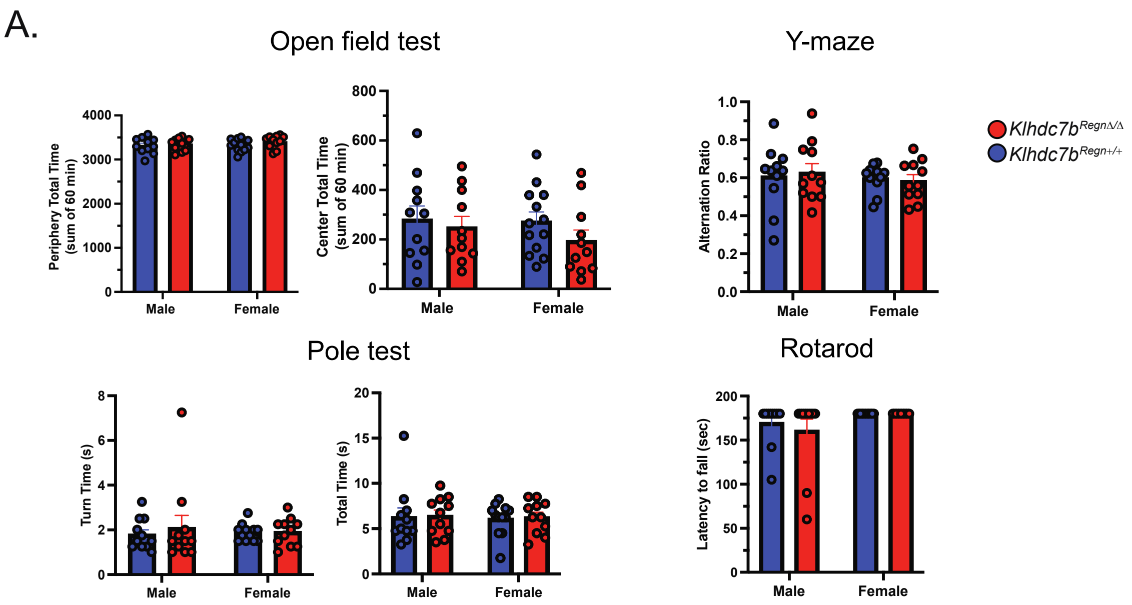

Supplementary Figure 3. Behavioral testing in *Klhdc7b*$^{Regn+/+}$, and *Klhdc7b*$^{Regn\Delta/\Delta}$ mice. For the open field, mice were 8-12 weeks of age. For the Y-maze, pole test and rotorod, mice were 12-16 weeks of age. Two-way ANOVA was performed and found no significant main effect of genotype or sex on any of the behavioral tests performed. n = 10 mice per sex per genotype. All error bars show standard deviation.

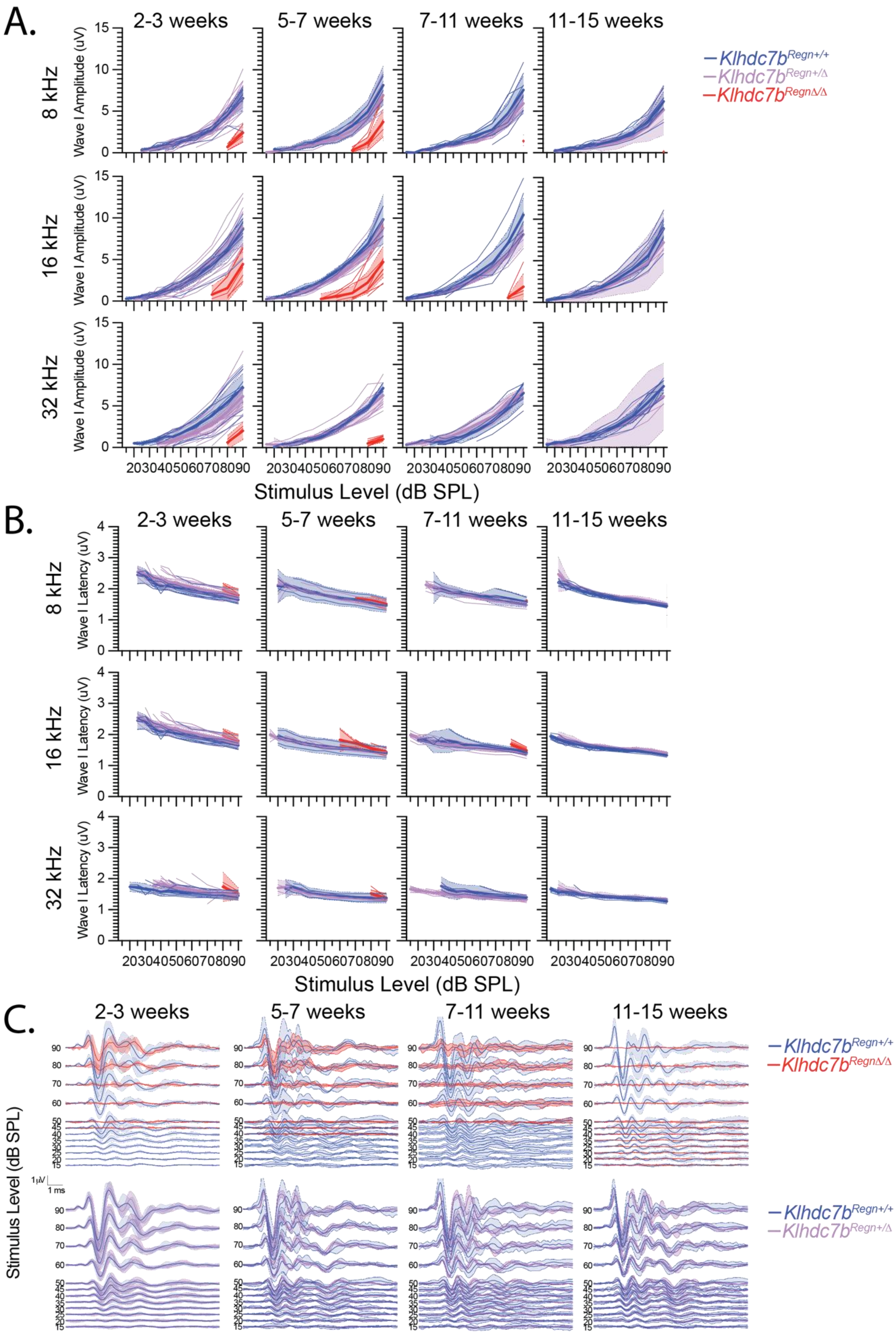

A.
2-3 weeks
5-7 weeks
7-11 weeks
11-15 weeks
8 kHz
16 kHz
32 kHz
Wave I Amplitude (uV)
Stimulus Level (dB SPL)
B.
Wave I Latency (uV)
C.
Stimulus Level (dB SPL)
1µV
1 ms
Klhdc7b Regn+/+
Klhdc7b Regn+/Δ
Klhdc7b RegnΔ/Δ

Supplementary Figure 4. Detailed time course recordings of *Klhdc7b*$^{Regn\Delta/\Delta}$, *Klhdc7b*$^{Regn+/\Delta}$, *Klhdc7b*$^{Regn+/+}$ mice at different time points. **A**. Wave I amplitudes for all time points and frequencies tested. Analysis was performed via two-way ANOVA, which found a significant main effect of genotype. **B**. Wave I latency for all time points and frequencies tested. No differences were significant. For A and B, thin lines represent individual mice, thick lines represent the mean, and shaded areas between dotted lines indicate 95% confidence interval. **C.** Average recordings at different time points, all of 16 kHz. Top row, *Klhdc7b*$^{Regn\Delta/\Delta}$ and *Klhdc7b*$^{Regn+/+}$. Bottom row, *Klhdc7b*$^{Regn+/\Delta}$ and *Klhdc7b*$^{Regn+/+}$. Shaded area is standard error of the mean. n=3-17 mice per group, mixed sex

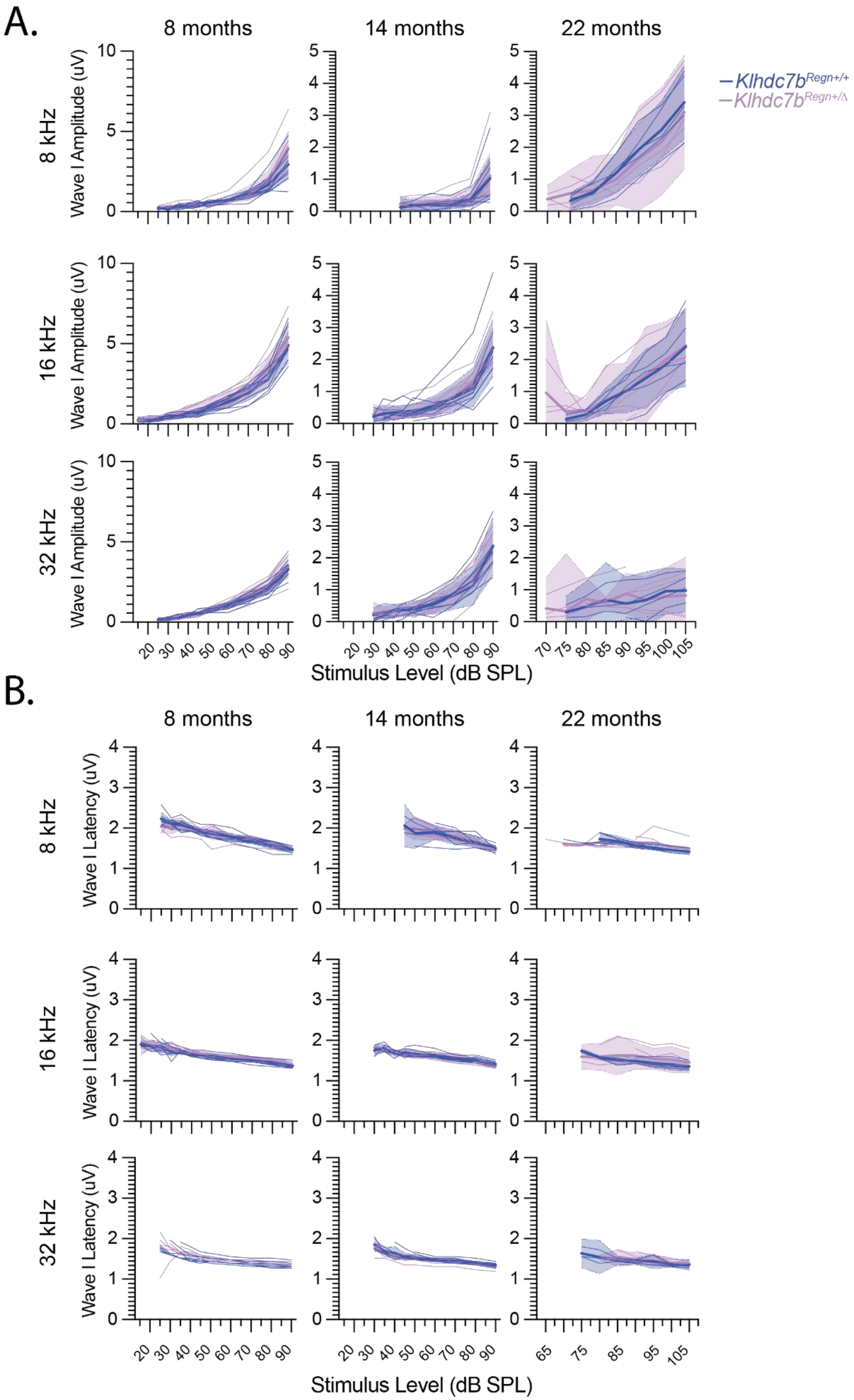
A.
8 months
14 months
22 months
8 kHz
16 kHz
32 kHz
Wave I Amplitude (uV)
Klhdc7b^Regn+/+
Klhdc7b^Regn+/Δ
Stimulus Level (dB SPL)
B.
8 months
14 months
22 months
Wave I Latency (uV)
Stimulus Level (dB SPL)

Supplementary Figure 5. A. Wave I amplitudes from longitudinal recordings of *Klhdc7b*$^{Regn+/\Delta}$ and *Klhdc7b*$^{Regn+/+}$ mice at 8 months (left column), 16 months (middle column) and 22 months (right column). **B.** Wave I latencies from longitudinal recordings of *Klhdc7b*$^{Regn+/\Delta}$ and *Klhdc7b*$^{Regn+/+}$ mice at 8 months (left column), 16 months (middle column) and 22 months (right column). For A and B, Data was analyzed by two-way ANOVA or mixed model and no significant differences between groups were found. Thicker lines indicate means, thin lines individual mice, and shaded areas between dotted lines are 95% confidence intervals. N = 5-8 mice per group, mixed sex.

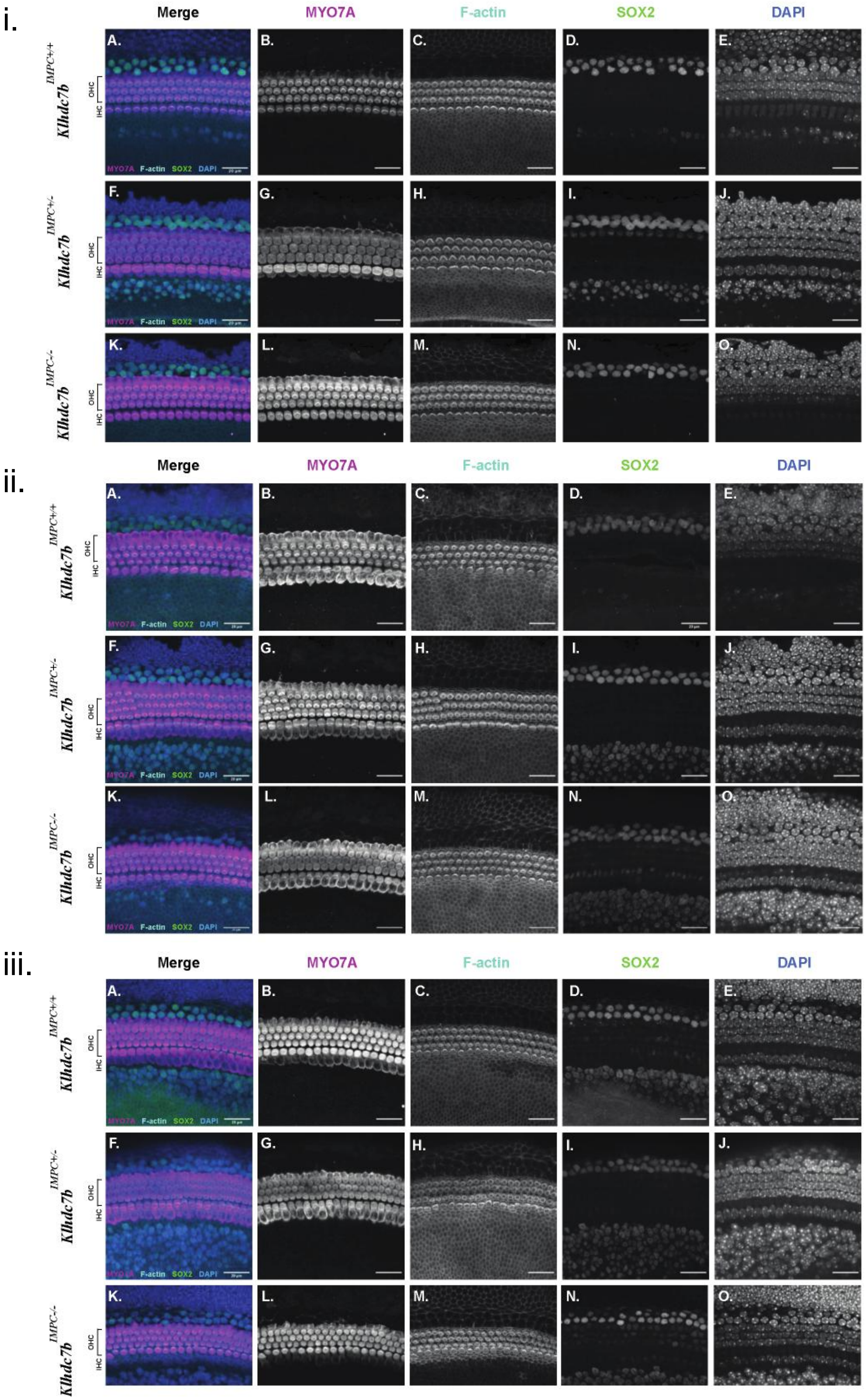

i.
ii.
iii.
Merge
MYO7A
F-actin
SOX2
DAPI
Klhdc7b IMPC+/+
Klhdc7b IMPC+/-
Klhdc7b IMPC-/-
OHC
IHC
A.
B.
C.
D.
E.
F.
G.
H.
I.
J.
K.
L.
M.
N.
O.
MYO7A F-actin SOX2 DAPI

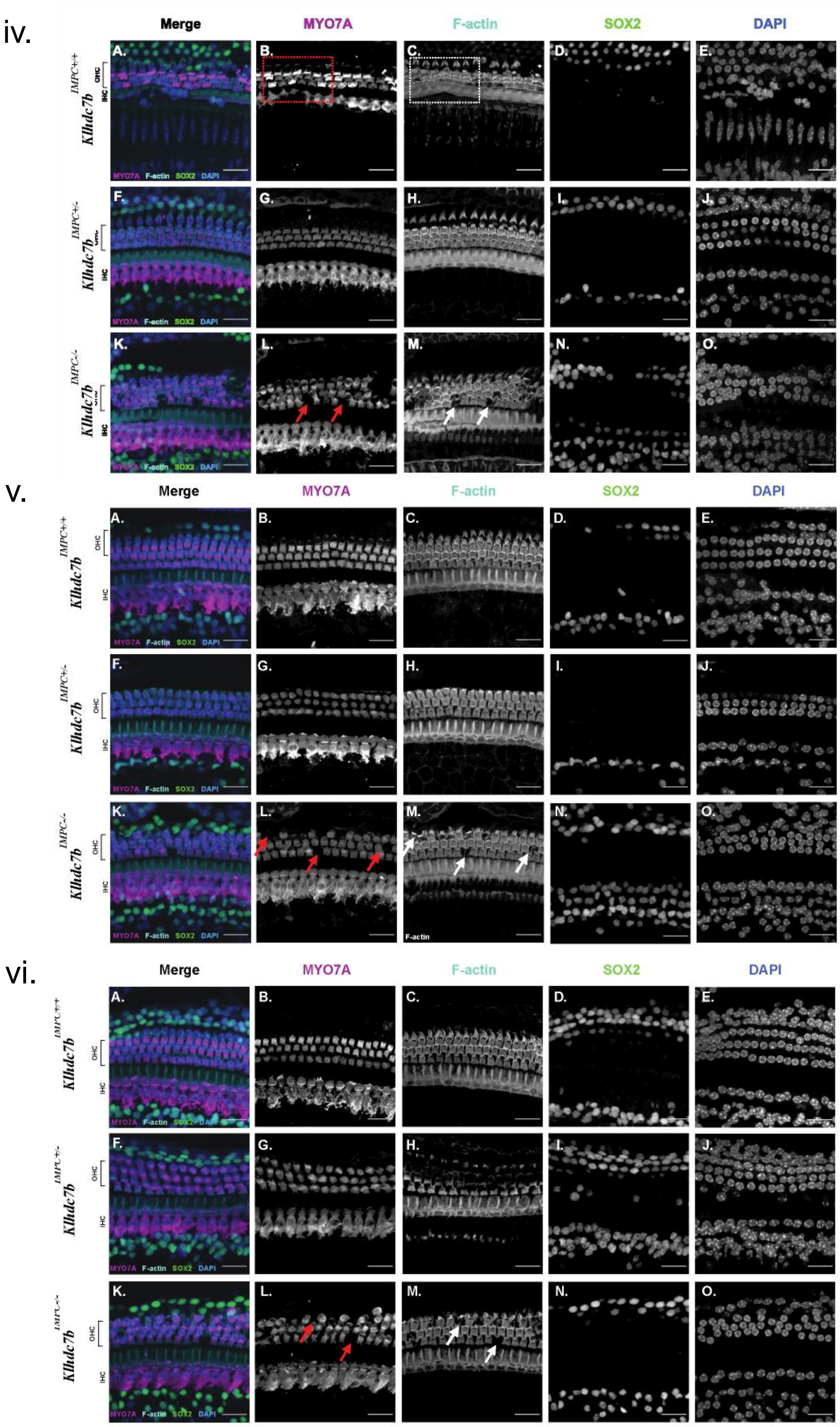
iv.
Merge
MYO7A
F-actin
SOX2
DAPI
Klhdc7b IMPC+/+
Klhdc7b IMPC+/-
Klhdc7b IMPC-/-
OHC
IHC
v.
Merge
MYO7A
F-actin
SOX2
DAPI
Klhdc7b IMPC+/+
Klhdc7b IMPC+/-
Klhdc7b IMPC-/-
OHC
IHC
vi.
Merge
MYO7A
F-actin
SOX2
DAPI
Klhdc7b IMPC+/+
Klhdc7b IMPC+/-
Klhdc7b IMPC-/-
OHC
IHC

vii.

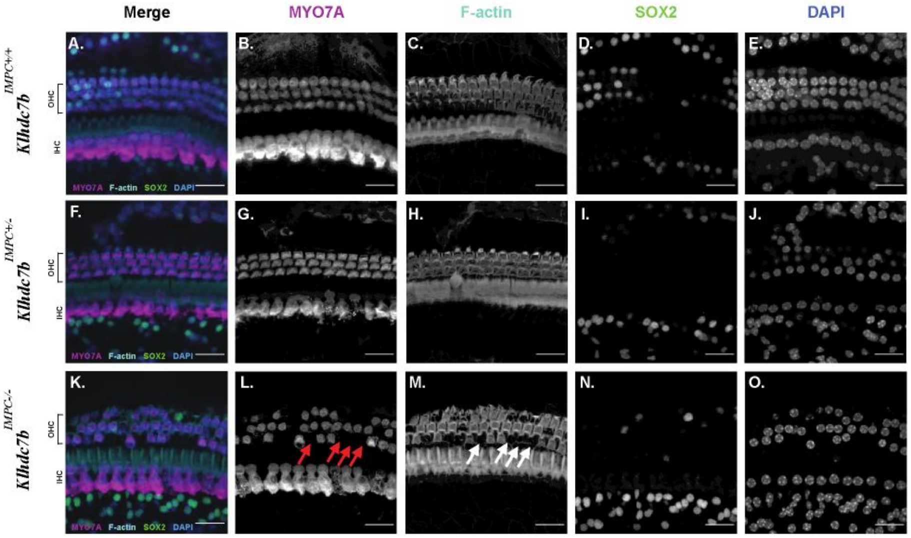
Merge
MYO7A
F-actin
SOX2
DAPI
Klhdc7b IMPC+/+
Klhdc7b IMPC+/-
Klhdc7b IMPC-/-
OHC
IHC
A.
B.
C.
D.
E.
F.
G.
H.
I.
J.
K.
L.
M.
N.
O.
MYO7A F-actin SOX2 DAPI

viii.

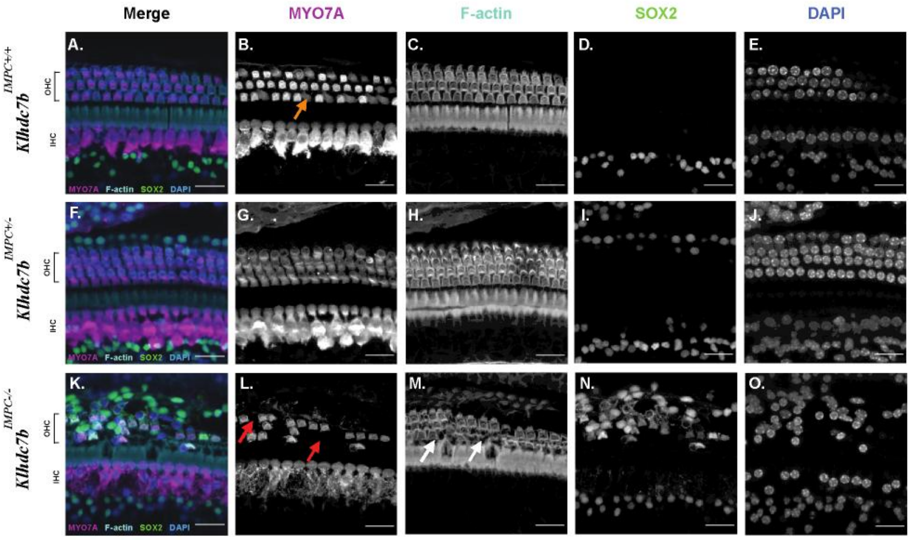
Merge
MYO7A
F-actin
SOX2
DAPI
Klhdc7b IMPC+/+
Klhdc7b IMPC+/-
Klhdc7b IMPC-/-
OHC
IHC
A.
B.
C.
D.
E.
F.
G.
H.
I.
J.
K.
L.
M.
N.
O.
MYO7A F-actin SOX2 DAPI

ix.

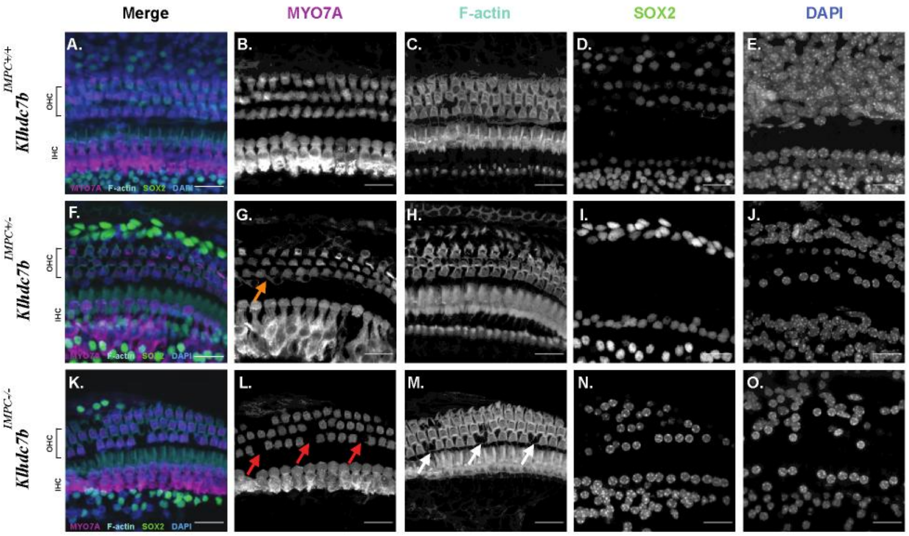
Merge
MYO7A
F-actin
SOX2
DAPI
Klhdc7b IMPC+/+
Klhdc7b IMPC+/-
Klhdc7b IMPC-/-
OHC
IHC
A.
B.
C.
D.
E.
F.
G.
H.
I.
J.
K.
L.
M.
N.
O.
MYO7A F-actin SOX2 DAPI

Supplementary Figure 6. Cochlea whole mounts are shown in panels (i) to (ix) from the basal, mid and apical turn of P2 - P4 (i-iii), 3 week (P19-P21, iv-vi) and 8 week (P58-P60, vii-ix) mice in $Klhdc7b^{IMPC+/+}$, $Klhdc7b^{IMPC+/-}$, $Klhdc7b^{IMPC-/-}$ mice. Hair cells immunolabelled using Myosin VIIa (MYO7A) in magenta, actin cytoskeletal and stereocilia bundles labelled using Phalloidin (F-actin) in cyan, non-sensory epithelium immunolabelled using Sox2 in green, and nuclear immunostaining with DAPI in blue. OHC = Outer hair cells (indicating the three rows of OHCs). IHC = Inner hair cell. Scale bar = 20 µm, all images representative of $n \geq 3$ mice per genotype. The three rows of OHCs can be seen in parallel to the single row of IHCs (Myosin VIIa), on either side supporting cells are labelled using Sox2. The IHC and OHC are labelled at the side of panels A, F, K. Red and white arrows indicate examples of OHC loss. Some artefactual damage as part of mounting is present in viii and ix: orange arrows indicate where hair cells may appear to be missing but they are just folded over. Red and white box in iv also indicate some artefactual damage on mounting.

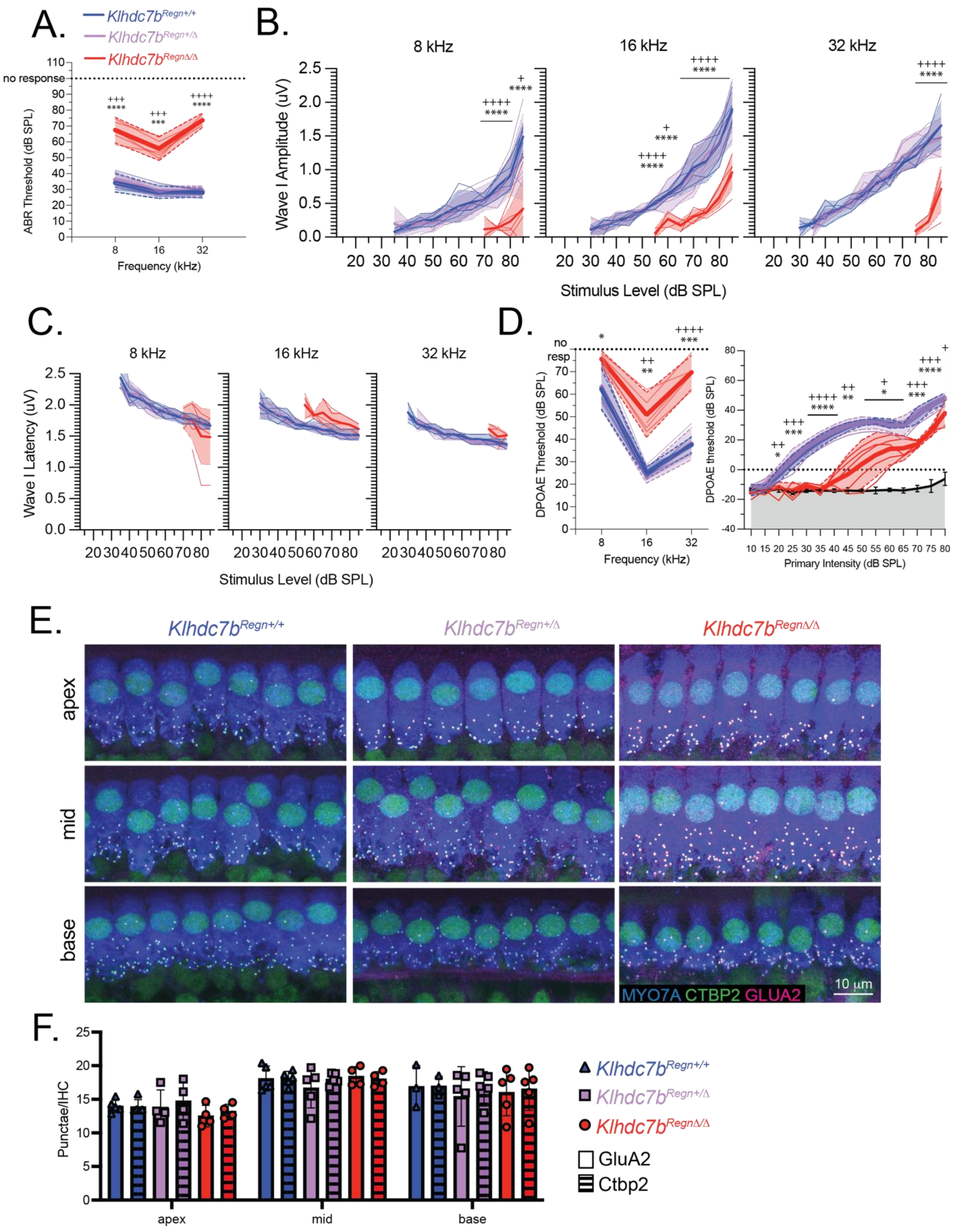
A.
Klhdc7bRegn+/+
Klhdc7bRegn+/Δ
Klhdc7bRegnΔ/Δ
no response
ABR Threshold (dB SPL)
Frequency (kHz)
B.
8 kHz
16 kHz
32 kHz
Wave I Amplitude (uV)
Stimulus Level (dB SPL)
C.
8 kHz
16 kHz
32 kHz
Wave I Latency (uV)
Stimulus Level (dB SPL)
D.
no resp
DPOAE Threshold (dB SPL)
Frequency (kHz)
DPOAE threshold (dB SPL)
Primary Intensity (dB SPL)
E.
Klhdc7bRegn+/+
Klhdc7bRegn+/Δ
Klhdc7bRegnΔ/Δ
apex
mid
base
MYO7A CTBP2 GLUA2
10 μm
F.
Punctae/IHC
apex
mid
base
Klhdc7bRegn+/+
Klhdc7bRegn+/Δ
Klhdc7bRegnΔ/Δ
GluA2
Ctbp2

64

Supplementary figure 7. Detailed analysis of ABR, DPOAE, and synaptic structures in a cohort of 3-week-old *Klhdc7b*$^{Regn\ \Delta/\Delta}$, *Klhdc7b*$^{Regn+/\Delta}$ and *Klhdc7b*$^{Regn+/+}$ mice. A. ABR thresholds of 3-week-old mice. **B.** ABR Wave I amplitude of the same mice. **C.** ABR Wave I latency of the same mice. **D.** DPOAE thresholds (left) and input/output curve at 16 kHz (right). Legend in A refers to A-D. For A-D, thin lines represent individual mice, thick lines represent the mean, and shaded areas between dotted lines indicate the 95% confidence interval. Analyzed by two-way ANOVA followed by Tukey's post-hoc tests. **E.** Examples of synaptic staining of apex (top row), middle turn (mid, middle row), and base (bottom row) inner hair cells stained against CTBP2 (presynaptic ribbon) and GLUA2 (postsynaptic AMPA receptor subunit) and MYO7A. **F.** Synaptic puncta were counted and normalized to the number of inner hair cells. No difference was found between genotypes. All error bars show standard deviation.

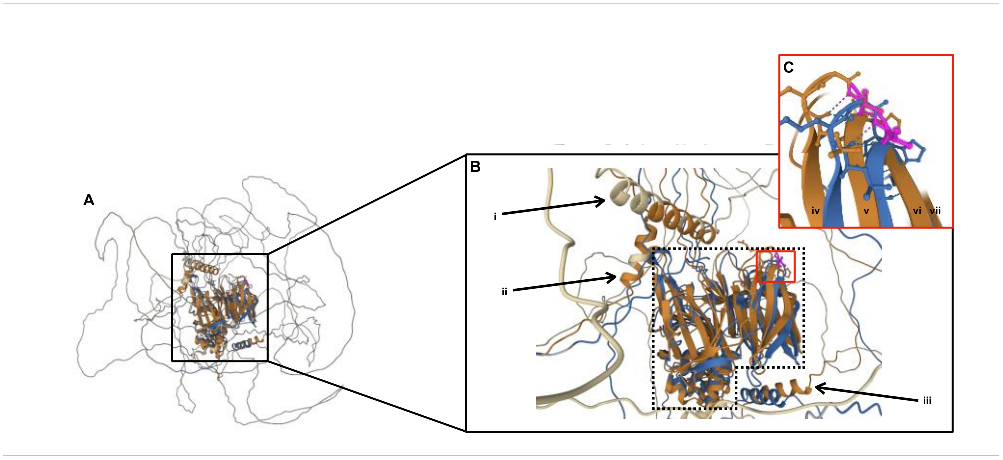

Supplementary Figure 8. Overlay analysis of the KLHDC7B long and short isoforms using the Pairwise structural alignment tool created by RCSB Protein Data Bank. **A.** Low resolution of both Klhdc7b isoforms, the long isoform (orange) and the short isoform (blue). The Kelch β-propeller of each variant is viewed in the same plane as the β-sheets. **B.** shows the KLHC7B proteins core at higher resolution and both the V1154 and V504 residues are annotated in magenta, (**i-ii & iii)** show $\boldsymbol{\alpha}$-helixes in close proximity with the Kelch β-propellers; however, i and ii are only within the long isoform structure and although iii is found in both isoforms, it can be seen that they do not appear to align completely. **C.** shows an enhanced resolution of the fifth Kelch motif in both isoforms, the valine residue is located within an inter-blade loop, and it can be seen than positions of the first two β-sheets of the long and short isoforms appear **(iv-v)** to be different but the third and fourth β-sheets β-propeller **(vi-vii)** in similar positions. This suggests that the Kelch β-propeller is subtly different between these two isoforms.

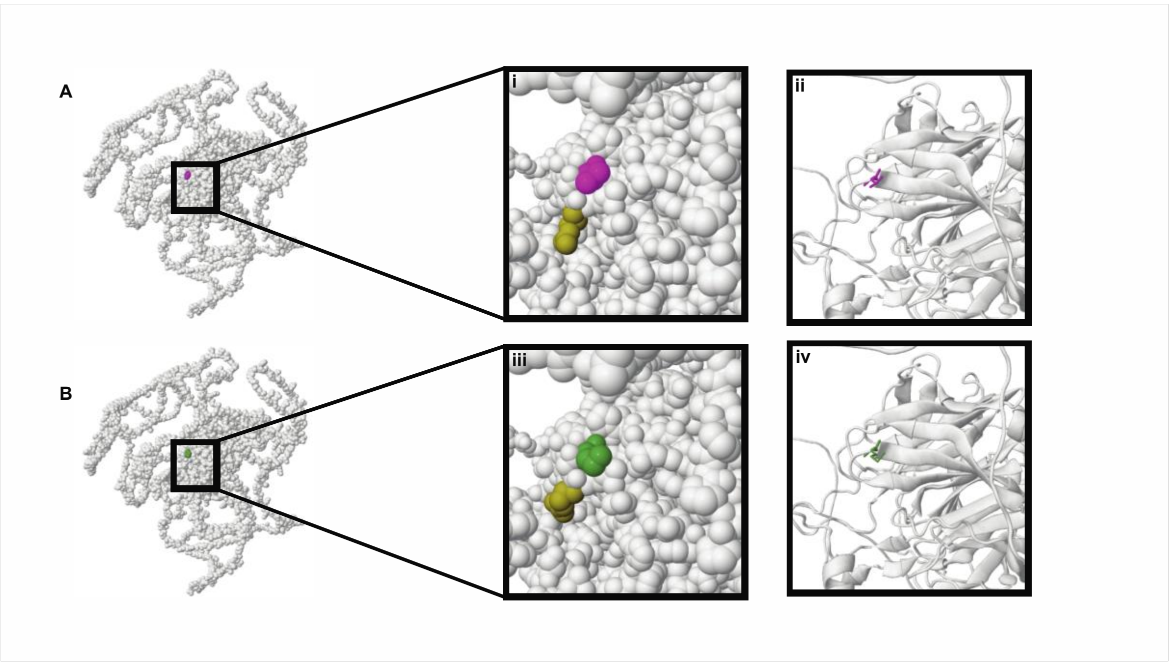

Supplementary Figure 9. Calotte space-filling modelling of Val and Met alleles on the long isoform of KLHDC7. Missense 3D modelling predicts the Val1154Met variant long is structurally damaging on the long isoform following contraction of cavity volume by 395.928 Å^3, the threshold for this criterion is an expansion or contraction of the cavity volume ≥70 Å^3 and it defines a cavity as either within the core regions of the protein or a pocket on the surface. **A.** KLHDC7B Val1154 (magenta) and **B.** KLHDC7B Met1154 (green). Boxes i and iii show the two residues at higher resolution and ii and iv show the same region at high resolution using as cartoon modelling. There is a subtle change in an adjacent space fill region (seen in yellow) following the missense mutation.

Supplementary Table 1. VEP predictions for the effect of the ARHL associated Val>Met variant on the short and long form of Klhdc7b.

SNP ID	Ensembl Transcript ID	Amino Acid	Position	SIFT	PolyPhen
rs36062310	ENST00000395676.4	V/M	504	Tolerated (0.06)	benign (0.278)
rs36062310	ENST00000648057.3	V/M	1154	Deleterious (0)	possibly damaging (0.812)

<u>Supplementary Methods</u>

qPCR probes

Species	Name, gene	Probe	Forward primer	Reverse primer	company
mouse	L+S<br>mouse<br>*Klhdc7b*	TTTATGCCATTG<br>GTGGCGAGTGC	GGTGGCCCTG<br>GATGGAATG	TCTGTGCGTG<br>GGTCATAGC	IDT<br>(Integrated<br>DNA<br>technologies)
mouse	L mouse<br>*Klhdc7b*	TCCCCATCATGA<br>T<br>CCACTTTACCCC	CCGCAAAGCAA<br>AGGATATTCCT<br>G	ATGCCTCCAG<br>CTGCATCTCT<br>A	IDT
mouse	Mm01310009<br>_<br>m1_Drosha,<br>Drosha				Thermofisher

RNA extraction

Tissue was homogenized in TRIzol®, and chloroform was used for phase separation. Total RNA was purified using MagMAX™-96 for Microarrays Total RNA Isolation Kit (Ambion by Life Technologies) according to manufacturer's specifications. Genomic DNA was removed using RNase-Free DNase Set (Qiagen).

Generation of *Klhdc7b*$^{RegnD/D}$ knockout mice

Summary description of sequences

**Description**	**Features**
Mus Musculus *Klhdc7b*, mRNA,<br>NM_001160178	Length: 5,764 bp<br>CDS: 859-4,650<br>Exons: 1-5,764;
Mus musculus Klhdc7b, protein	Length: 1,263 aa
LacZ replacing *Klhdc7b,* DNA	Length: 3,075 bp
LacZ replacing *Klhdc7b,* protein	Length: 1,026 aa
5' mouse UTR // **Start**, **<u>Acc65</u>** // 5' LacZ	5'mouse // 5' LacZ
3' LacZ //**Stop** // (*<u>LoxP</u>*) // **NheI** // 3' mouse	3' LacZ, 3' mouse UTR
Primers and probes for loss of allele and gain of allele assays for *Klhdc7b* KO	Table 1

Table 1

**NAME**	**Primer**	**Sequence (5'-3')**
4929mTU	Forward<br>Probe (BHQ)<br>Reverse	TGGGAGGCGGTTGTACTC<br>ACCTTGCTGGCACTAGCGGTGGT<br>GCAAAGCCAGTGCTAAGGT

4929mTD	Forward<br>Probe (BHQ)<br>Reverse	GGCTTCCTATACCGCTTTGAC<br>TCCAGCTATCGCACTTATCTGA<br>CTCCAGGAGCCGGTCACT
LacZ	Forward	GGAGTGCGATCTTCCTGAGG
	Probe (BHQ)	CGATACTGTCGTCGTCCCCTCAAACTG
	Reverse	CGCATCGTAACCGTGCATC
Neo	Forward	GGTGGAGAGGCTATTCGGC
	Probe (BHQ)	TGGGCACAACAGACAATCGGCTG
	Reverse	GAACACGGCGGCATCAG

Description of BAC clone generation for Klhdc7b$^{RegnD/D}$ mice

A BAC clone RP23-241G24 containing a mouse *Klhdc7b* gene was used and modified as follows. Briefly, a DNA fragment was generated to include a mouse 5' homology nucleotide sequence of 100 bp (mHU), a LacZ gene (3,075bp) downstream and in frame with the ATG starting site of *Klhdc7b* gene, followed by a self-deleting neomycin cassette of 4,809 bp, and a 3' mouse homology sequence of 100 bp (mHD). This DNA fragment was used to modify BAC clone RP23-241G24 through homologous recombination in bacterial cells. As result, a full KO of the region encoding mouse *Klhdc7b* genomic fragment of 3,787 bp in the BAC clone was replaced by the LacZ, a Neo-self-deleting cassette (SDC) of 8,202 bp. The entire mouse *Klhdc7b* orf (mm9, chr15:89,215,351-89,219,137) was replaced with the LacZ-Neo SDC leaving intact 5' and 3' UTRs. The resulting modified BAC clone included, from 5' to 3', (i) a 5' mouse homology arm containing about 140.5 kb of mouse genomic DNA including a mouse *Klhdc7b* 5' UTR and ATG; LacZ cDNA of 3,075 bp, a self-deleting Neomycin cassette of about 4,809 bp, followed by a 3' mouse homology arm of 12.6 kb containing the mouse *Klhdc7b* 3' UTR and the remaining mouse genomic DNA in the original BAC clone.

The modified BAC clone containing the KO of *Klhdc7b* gene, LacZ and a self-deleting Neomycin cassette was used to electroporate mouse embryonic (ES) cells to create modified ES cells comprising a *Klhdc7b* KO gene into B6 mice and content confirmed by TaqMan assay. Female mice were implanted using the VELOCIMOUSE® method (see, e.g., U.S. Pat. No. 7,294,754). Briefly, ES cells were electroporated into an early-stage blastocyst (8 cells Morula stage) to generate first generation (F0) mice allowing immediate phenotypic analyses[1]. The Neomycin selection cassette was removed by crossing the progeny generated from the ES clone with a deletor rodent strain that expresses a Cre recombinase.

Primers for Klhdc7b$^{IMPC-/-}$ mouse genotyping

**Primer name**	**Sequence 5' → 3'**	**Primer Type**
Klhdc7b-DEL1258-UNI_F	CCTCTGAAGGCGCCATCTTC	Universal Forward
Klhdc7b-DEL1258-WT_R	TGCTTGGTTTCATCCCCTCTC	Wildtype Reverse
Klhdc7b-DEL1258-MUT_R	CCTAGCGCCACCATTCACTG	Mutant Reverse

Antibodies and Stains

**Antibody Name (target)**	**Source**	**Catalog**	**primary/ secondary**	**host species**	**dilution**
Myo7A	proteus	25-6790	primary	rabbit	1 in 1000
KLHDC7B Immunogen: entire short isoform of human protein	custom	Custom	primary	rabbit	1 in 200-600
Sox2	BDPharmingen	AB_1645334	primary	mouse	
Ctbp2 (IgG1)	BD Transduction Labs	612044	Primary	Mouse	1 in 200
GluA2 (IgG2a)	Millipore	MAB397	Primary	Mouse	1 in 1000
DAPI	Thermofisher	62248	cell stain	N/A	1 in 1000
Alexa Fluor 647 phalloidin	Thermofisher	A22287	cell stain	N/A	1 in 500
phalloidin-Atto 647N	Sigma	# 65906	cell stain	N/A	10nM
donkey anti-rabbit Alexa 488	Thermofisher	A32790	secondary	donkey	1 in 1000
donkey anti-mouse Alexa 568	Thermofisher	A10037	secondary	donkey	1 in 1000
goat anti-rabbit (IgG H+L) Alexa 488	Thermofisher	A-11008	secondary	goat	1 in 1000
goat anti-(mouse IgG2a) Alexa 546	Thermofisher	A-21133	secondary	goat	1 in 1000
Goat anti-mouse IgG1 Alexa 568	Thermofisher	A-21124	secondary	goat	1 in 1000
Goat anti-mouse IgG2a Alexa 488	Thermofisher	A-21131	secondary	goat	1 in 1000

RNA scope reagents

**Reagent**	**Source**	**Catalog/reference #**
Mouse *Klhdc7b* long probe only	ACD bio	1088961-C1
Mouse *Klhdc7b* overlapping probe	ACD bio	1136591-C2
Mouse positive control probes	ACD bio	320881
negative control probes	ACD bio	320871
protease and peroxide kit	ACD bio	322381
fluorescence detection kit	ACD bio	323110
Opal 520	Akoya biosciences	OP-001001
Opal 570	Akoya biosciences	OP-001003
Anti-MYO7A antibody	Proteus	25-6790
Donkey anti-rabbit Alexa 647	Thermofisher	A31573
Prolong gold	Thermofisher scientific	P36934

Behavioral testing

One week before starting behavioral evaluations mice were handled for five minutes over two consecutive days to reduce the impact of handling on behavioral readouts. On experiment day, mice were additionally acclimatized to the experimental room at least for an hour.

Open Field. Mice were placed in an open field Plexiglas arena (40.6 cm x 40.6 cm x 38.1 cm) containing two horizontal laser beam detection arrays. The tracking software Motor Monitor (Kinder Scientific) for Windows (Microsoft) was used to measure movement in X and Y axis. Groups of eight animals, counterbalanced across experimental conditions, were tested concurrently for 60 minutes. Behavior was measured in five-minute intervals and a total count for each measure for the 60 minutes was calculated by summing the twelve five-minute bins. Computed measures include: basic movements (any horizontal beam cross), immobility time (lack of horizontal and vertical beam crosses), fine movements (changes in body position not meeting criteria for ambulation, includes grooming and head movements), X+Y axis ambulation (complete relocation of the animal's body), rears (vertical beam crosses), rearing time (time spent breaking vertical beams), rest time (lack of beam crosses lasting longer than 15 seconds), and total distance traveled (computed from known distances between beams and total beam crosses).

Rotorod. Animals were trained to walk on the Rotorod equipment (IITC Life science) containing 5 separate lanes with individuals rotating drums. Training consisted of 3 separate runs of 180 seconds where rotation speed was progressively increased: trial 1 (0 to 15 rpm, rotations per minute), trial 2 (7 to 15 rpm) and trial 3 (7 to 15 rpm). Mice were replaced back onto drum if they fell off. An animal was considered ready for testing phase if able to walk on the drum for at least 150 seconds. Three testing trials per animal were run (inter-trial interval = 30 minutes). During testing trials rotation speed was 15 rpm and mice were assessed for 180 seconds until a fall occurred. The median latency to fall was computed for each mouse.

Pole Test. The pole test was carried on a metal rod (50 cm long, 1 cm diameter) mounted on a square base that was buried under home cage flooring. During adaptation trials each mouse was placed three times on the rod (head down) to facilitate learning of pole-descent. In testing trials mice were placed on the top part of the rod (head up) and evaluated for the ability to turn around and descend the pole safely. The time to make a full 180 degrees turn and latency to reach the floor was recorded for 5 consecutive times. Trials where mice fell off the rod were annotated with the maximum trial duration (60 seconds). Average time to turn around and descend were calculated for the best 4 (out of 5) consecutive trials.

Y-maze (spontaneous alternation). Mice were placed in a Y-maze apparatus constituted of three plastic arms (14.5 cm × 3.5 cm × 13.5 cm) separated by a 120° angle from each other. At the beginning of each trial mice were positioned at the center of the maze and allowed to explore for 8 minutes. Using Ethovision system (Noldus, The Netherlands) body position was tracked over time to compute the number of spontaneous arm alternations (visits to an arm that was previously not visited) and the maximum number of possible alternations after a given number of visits to a new arm. Alternation ratio (AR) was calculated by dividing spontaneous arm alternations over maximum number of possible alternations.

ABR

For recordings at Regeneron, equipment was calibrated each day using a microphone (model PCB 378C01, PCB Piezotronics, NY) placed at the same distance from the speaker as the mouse

ear being recorded. Animals were anesthetized with an intraperitoneal injection of ketamine/xylazine (12 mg/kg, 0.5 mg/kg) and placed in a heated cage. Puralube ointment was placed on the eyes after several minutes once the animal was no longer responsive. Once the mouse was fully anesthetized it was placed on a Gaymar heating pad (Gaymar Industries, NY) in the sound booth. Electrodes were plugged into a Medusa 4Z preamplifier (Tucker-Davis Technologies, FL). Three lead, 13mm needle electrodes were placed subdermally with the lead electrode at the cheek of the animal (near the cochlea), the reference electrode at the midline of the skull on top of the head, and the ground electrode in the contralateral cheek. The ear being recorded was positioned 7.5 cm away from the speaker, in an open field configuration. After recordings, animals were placed in a heated recovery cage and returned to the home cage once ambulatory. For recordings at Harwell, mice were anesthetised by intraperitoneal injection of ketamine (100 mg ml^{-1} at 10% v/v) and xylazine (20 mg ml^{-1} at 5% v/v) administered at the rate of 0.1 ml per 10 g body mass. Animals were placed on a heated mat inside a sound-attenuated chamber (ETS Lindgren) and electrodes were placed subdermally over the vertex (active), along the right mastoid (reference) and on the left flank (ground). Animals were recovered using 0.1-0.2 ml of anaesthetic reversal agent atipamezole (5 mg ml^{-1} at 1% v/v).

ABR analysis

For automated threshold calling, the covariance between pairs of adjacent decibel traces was calculated and plotted. These points were fit to a curve using a sigmoid or logarithmic function, and the decibel level at which the function crossed below a set criterion level was recorded as the threshold of hearing. The threshold values called by the algorithm were compared to manually called thresholds. Traces with no discernible ABR response were called at 100 dB. If the difference between the manual and automated thresholds was greater than 15 dB, traces were examined and the manual threshold was used; otherwise, the automated threshold was used. For wave 1 amplitude and latency, peaks were detected using a semi-automated method where a peak and trough was estimated within a time window to encompass wave 1, then checked by a user and corrected if necessary.

ABR and DPOAE for Supplementary Figure 7

ABR and DPOAE were measured with an apparatus containing two speakers, probe tubes and a probe tube microphone which was based on designs from the Eaton-Peabody Labs at the Massachusetts Eye and Ear Infirmary, called the DPOAE starship[2,3]. ABR thresholds were called as in Shaheen et al., 2024[4]. DPOAE recordings were measured for stimuli at 8, 16, and 32 kHz pure-tone frequencies. Two primary tones ($f1$:$f2$ = 1.2) were presented at each with the $f1$ primary tone 10 dB higher than $f2$. The f2 stimulus was presented starting at 10 dB SPL and increasing in 5 dB steps up to 80 dB. Each stimulus frequency and level was averaged across 100 repeated measurements. DPOAE thresholds were defined as the f2 level required to produce a cubic distortion tone ($2*f1 - f2$) of 5 dB SPL. The sensitivity of the microphone was calibrated *in situ* to be within 2 dB of the target level before each recording.

3D microscopy

Staining and clearing protocol. Cochlea were collected, stored in PBS, and decalcified with immunocal overnight, then rinsed 3x with PBS at room temperature. All washes and incubations were conducted with shaking. The decalcified bone was carefully cut around the spiral to

improve reagent penetration. Cochleae were washed 3x 2 hours with PBS at room temperature, then blocked for two hours at 37°C, using standard blocking solution as for immunohistochemistry. MYO7A antibody was diluted in blocking solution at 1:200-1:300, then samples were incubated in primary antibody for 48-72 hours at 37°C. Samples were washed 3x for two hours in PBS at room temperature. Secondary antibody (Donkey anti-rabbit Alexa 568, thermofisher, A10042) and a nuclear stain (Sytox Deep Red, Thermofisher, S11381) were diluted at 1:200 and 1:400, respectively, in blocking solution. Samples were incubated in this solution overnight at 37°C, then washed 3x for two hours in PBS at room temperature. Samples were incubated at room temperature in a dilution series of concentrations of methanol (30% methanol, 50%, 70%, 90% and 98%) plus 2% Tween-20 in ddH_2O for 8-18 hours each. Samples were then placed in a glass jar and incubated at room temperature in 100% Ethyl Cinnamate (ECi) for two hours, then placed in fresh ECi and incubated at room temperature for ~72 hours. Samples were stored in ECi at room temperature or at 4°C until imaging.

Imaging. Samples were imaged using an UltraMicroscope Blaze (Miltenyi Biotec). The 12x objective was used with the 1.67x tube lens for a total magnification of 20x with 100% beam width and beam thickness of 3.9um, with illumination from both sides and five steps of dynamic focus. Z stacks were spaced at 2 mm. Samples were imaged using the 561 and 640 lasers for Alexa 568 and Sytox deep red, respectively.

Automated Hair cell counting in intact cochlea

Virtual Dissection. After imaging, files were processed and converted to Imaris (v10.1.0) files. To minimize the need for large computational resources, images were cropped in 3D to focus solely on the hair cells. Hair cells were virtually dissected using the 'filament' tool in Imaris by tracing the inner hair cells from the apex to the base as described by Hutson et al[5]. The filament diameter was set to 120 mm to capture both inner and outer hair cells. To create a mask, the Imaris 'Filament to Channel' Xtension was used to generate a new channel of data based on the filament. The channel was converted using the 'Surface' tool in Imaris based on the signal from the new filament channel. The generated surface was used to mask the original data channels containing both nuclear signal and hair cell fluorescence. Data from both masked channels were exported as TIFF images (along the *z*-axis) for automatic counting of cells. In addition to masking the combined signal from the inner and outer hair cells, an identical procedure was employed to isolate signal from only the inner hair cells. All subsequent analysis was performed using Python (v3.9.6) on a 32-core cloud-based compute cluster with 128 GB of RAM.

Automated Hair Cell Counting. Masked hair cell nuclear and fluorescence images were tiled into 150 x 150 pixel^2 sub-images to further maximize processing on data in the limits of available memory during automated counting (I_{nuc} , I_{FLR} ,respectively). Simultaneously, an outer hair cell mask (M_{OHC}) was generated using the inner hair cell mask (M_{IHC}) by:

1. Smoothing each *z*-slice image via morphological closing
2. Radially offsetting the boundaries of inner hair cell mask by 100 pixels from the center of the cochlea spiral.
3. Morphological dilation of the offset mask by a disk (radius = 45 pixels)

For all further analysis, both the inner and outer hair cell masks were tiled in the same manner as the original nuclear and fluorescence images. To isolate hair cells, the masks for inner and outer hair cells were multiplied against the tiled nuclear and fluorescence images to extract pixels within each image that corresponded to either cell type (M_{IHC} x I, M_{OHC} x I). Hair cell fluorescence images were thresholded to exclude background fluorescence ($I_{FLR,\ thresh}$). After

excluding background fluorescence, we calculated the standard deviation per *z*-slice in each tile, and used local maxima per tile to identify *z*-slices of interest, including additional *z*-slices defined as z-slices within the full-width-at-half-maximum (FWHM) of the local maxima peaks. The identified *z*-slices were then processed individually to extract the hair cells using a hair cell selection function.

Briefly, a multi-level (four level) Otsu threshold was used to generate an image with four discrete pixel values from which the highest-valued pixels were isolated for both the nuclear and the fluorescence images per *z*-slice. A four level Otsu threshold was selected based on the distribution of signal in both the nuclear and fluorescence images. As the hair cell fluorescence and nuclear signal overlapped maximally at the center of the hair cell, it was assumed that the product would isolate hair cell signals. Therefore, the product of the maximal intensity value was calculated to extract the hair cell signal (I_{nuc} x $I_{FLR, thresh}$). To further remove contribution of signal from nearby cochlear supporting cells that overlapped with the hair cell fluorescence, we removed smaller objects using an area threshold (<50 pixels^2 or ~ 5.3 mm^2).

To generate an individual hair cell mask, we calculated the distance between each pixel in a cell and the background. We then isolated the location of the maximum distance per cell (at the center of the cell) and marked the center of each hair cell. At each location (cell center), a uniform circle was drawn around the cell nucleus and used to label hair cell containing pixels in the hair cell signal images. To label and mask each individual cells in the *z*-slice, we applied a watershed algorithm to this image. At each labelled cell's centroid, the intensity was recorded for individual cell mask generation. A circle was then drawn from the centroid with a radius equal to the distance at which the signal per each cell decreased below 95% of the previously recorded centroid intensity. To ensure that cells were not counted more than once, each cell was compared to detected cell objects in the prior 10 slices and overlapping cell objects were removed. For each tile, we multiplied the labelled cell objects with the original masked cell image (I_{nuc} x $I_{FLR, thresh}$). This process was performed for each cell type (IHC and OHC). Once all tiles had been processed, all tiles were stitched back together to create a final XYZ stack (I_{IHC}, I_{OHC}, respectively).

All processing up to this point used 3D datasets, ensuring the preservation of positional relationships of all hair cells in three-dimensional space. To accurately count the hair cells from apex to base, a custom processing pipeline was developed to unwrap the cochlea in a sequence that reflects its natural configuration. Despite meticulous efforts used during tissue embedding, the alignment of cochleae with the global axis was not perfect. To compensate for this, each *z*-slice was analyzed sequentially, with cells object being counted as they became visible and positionally stored in a growing map. When newly-visualized cell objects appeared more than 100 pixels from the existing cell object, or when cell objects appeared to be unwrapping in two directions, a new map was initiated to track the "far" or "diverging" cell objects.

After mapping the entire cochlea in 3D, the center of the maximum intensity projection (MIP) image (along the *z*-axis) was used to select one of the maps created above as the starting point to unwrap the cochlea. To select the starting map, we calculated the minimum distance between the center of the MIP and the *(x,y)* positional coordinates of all the maps we had generated. Once the start map (L_1) was identified, the map was traced to its opposite end, and the rotational direction from the center of the MIP was identified (either clockwise or counterclockwise) based on the change in the angle of rotation from the start to the end of the map. Subsequently, the remaining maps were examined to determine if the starting map (L_1) was next to another map in 3D space or was connected to either the base or apex of the cochlea. If a nearby map was identified near

the terminal end of L_1, the nearest point of this new map was linked to the L_1 map. This process was repeated until the final map reached either the apex or base of the cochlea.

After unwrapping part of the cochlea, the script would return to the start map of L_1 (middle of the cochlea) and reverse the rotational direction. It then searched for the nearest map to the starting point in 3D space, tracing the maps until reaching the opposite end of the cochlea (either apex or base). Upon completion of this process, the shorter of the two linked maps (assumed to contain the apex due to its length) was flipped such that the last map added became the starting point and the start map of L_1 was the end point. This map was then merged with the longer of the two linked maps (assumed to contain the base). This resulted in a naturally ordered mapping of the cochlea from the apex to the base. Simultaneously, accumulation of both inner and outer hair cells counts were traced along the finalized mapping, and cochlea length was calculated.

Subsequently, a tonotopic map of the cochlea was constructed using the outer hair cell length from the apex to the base[6]. Tonotopic mapping was calculated using:

$$freq\ (kHz) = 10^{\frac{156.5-(100-d)}{82.5}}$$

$$d\ (\%) = \frac{distance\ from\ apex\ (mm)}{6\ mm} \cdot 100$$

The normalization factor of 6 mm was selected based on the reported average length of a mouse cochlea from apex to base[7].

Results for all 12 cochleae were aggregated by excluding areas with damage to hair cells before assessing the rate of change in inner and outer hair cell counts. As the counting of cells was automated, individual IHCs were not mapped to corresponding rows of OHCs making instantaneous assessment of OHC to IHC ratio challenging. To account for this, we instead assessed the rate of change in OHC count and IHC count over discrete bins of cochlea length to extract an averaged ratio of OHC to IHC counts. The OHC to IHC ratio was calculated using 1 mm bins with ranges defined as 0 to 1 mm, 1.01 to 2 mm, 2.01 to 3 mm, 3.01 to 4 mm, and 4.01 to 5 mm. Through the use of binning and tracking rate of change in counts of each cell, removed the need to map each IHC to a corresponding row of OHCs while still providing a meaningful assessment of OHC to IHC ratio.

Cochlear explant cultures

Collagen bubbles were prepared by mixing 1.7 ml 1 N NaOH, 10 ml 10x PBS, 67 ml rat tail collagen, 21.3 ml H_2O. 10 ml of this solution was placed on the coverslip and cured at 37°C in a humidified incubator with 5% CO_2 for 30-40 minutes, then covered in PBS stored at 4°C.

Explant culture media was prepared as follows: 90 ml DMEM/F12 without phenol red, 7 ml fetal bovine serum, 1 ml penicillin G, and 1 ml L-glutamine.

GtTR conjugation

Gentamicin Sulfate salt (300 mg, Sigma Aldrich) in 6 mL of distilled water and triethylamine (0.17 mL, Sigma Aldrich) was added to a 15 ml vial. A solution of Texas Red N-Hydroxysuccinimide ester (AAT Bioquest) in N,N-Dimethylformamide (Sigma Aldrich, 1 mL, 50 mg/mL) was added slowly into the solution and agitated overnight at room temperature. The reaction solution was purified by reverse phase column chromatography using a Teledyne ISCO column, with a mobile phase consisting of Buffer A = 0.1% TFA in water, Buffer B = 0.1% TFA

in acetonitrile, and a gradient elution of 5-90% over in 20 min. LCMS gave a purity >90% and an observed m/z correct mass ion for the TFA salt, 1263 (M+TFA).

Whole mount and synaptic staining and quantification

Whole mount cochleae were dissected, the apex and round window membrane pierced, and post-fixed for 1 hour in 4% PFA at room temperature with gentle shaking, followed by 3 washes in PBS, decalcification in 4% EDTA for several days, then 3 washes in PBS and dissection. PBS was removed and samples were placed in 30% sucrose for 15 min at room temperature until pieces sank to the bottom of the well. Staining dish with sample was placed on dry ice until sucrose was completely frozen, then thawed at room temperature, optionally placed in a dish of room temperature water to hasten. Once thawed, sucrose was removed and samples were washed with PBS for 15 min with shaking at room temperature. Samples were then washed with 50 mM glycine (in PBS) for 30 min at room temperature, then treated with 10 mM sodium citrate (in distilled water, pH 8.5), at 65 ˚C for 20 minutes with shaking. 3x 10 min washes in PBS at room temperature with shaking were performed. Samples were blocked in 5% normal horse serum with 0.5% Triton X-100 for 1 h at room temperature, shaking. Primary antibodies against Ctbp2, GluA2 and MYO7A (or KLHDC7B and Prestin for Fig. 1E-F) were incubated overnight at room temperature with shaking in 1% normal horse serum with 0.5% Triton X-100 in PBS, followed by 3x 10 min washes in PBS at room temperature with shaking. Secondary antibodies in 1% normal horse serum with 0.5% Triton X-100 in PBS were incubated for 1 hour at room temperature with shaking, followed by 3x 10 min PBS washes at room temperature with shaking. Then the samples were incubated in an identical secondary solution for 1 hour at 37 ˚C. Cells were washed 3x 10 min PBS at room temperature with shaking, then mounted using prolong diamond with a high precision #1.5 coverslip (Zeiss) and imaged on an LSM 980.
Images were centered around inner hair cells, with a z stack taken encompassing the entire cell. ~3 fields of view per segment per animal were imaged. Quantification was performed in Imaris using surfaces and manually determining thresholds based on size and clarity above background.

References

1 Poueymirou, W. T. *et al.* F0 generation mice fully derived from gene-targeted embryonic stem cells allowing immediate phenotypic analyses. *Nat Biotechnol* **25**, 91-99, doi:10.1038/nbt1263 (2007).

2 Buran, B. N. *et al.* Optimizing Auditory Brainstem Response Acquisition Using Interleaved Frequencies. *J Assoc Res Otolaryngol* **21**, 225-242, doi:10.1007/s10162-020-00754-3 (2020).

3 Kim, Y. *et al.* Otof gene transfer in DFNB9 mice carrying human founder non-truncating alleles. *Genes Dis* **12**, 101590, doi:10.1016/j.gendis.2025.101590 (2025).

4 Shaheen, L. A., Buran, B. N., Suthakar, K., Koehler, S. D. & Chung, Y. ABRpresto: An algorithm for automatic thresholding of the Auditory Brainstem Response using resampled cross-correlation across subaverages. *bioRxiv*, doi:10.1101/2024.10.31.621303 (2024).

5 Hutson, K. A., Pulver, S. H., Ariel, P., Naso, C. & Fitzpatrick, D. C. Light sheet microscopy of the gerbil cochlea. *J Comp Neurol* **529**, 757-785, doi:10.1002/cne.24977 (2021).

6 Muller, M., von Hunerbein, K., Hoidis, S. & Smolders, J. W. A physiological place-frequency map of the cochlea in the CBA/J mouse. *Hear Res* **202**, 63-73, doi:10.1016/j.heares.2004.08.011 (2005).

7 Montgomery, S. C. & Cox, B. C. Whole Mount Dissection and Immunofluorescence of the Adult Mouse Cochlea. *J Vis Exp*, doi:10.3791/53561 (2016).
