# Supplementary material for: Cross-species validation of a human age-related hearing loss candidate KLHDC7B as essential for mammalian hearing
Source: Commun Biol. 2025 Dec 17;9:84. doi: 10.1038/s42003-025-09349-1 (PMC12820229; doi:10.1038/s42003-025-09349-1)
Supplement: Supplementary file 2 — Description of Additional Supplementary Files [file 42003_2025_9349_MOESM2_ESM.pdf]

Description of Additional Supplementary Files:

File: Supplementary Data 1

Description: The source data & statistics behind the graphs in the paper
