# Supplementary material for: Cross-species validation of a human age-related hearing loss candidate KLHDC7B as essential for mammalian hearing
Source: Commun Biol. 2025 Dec 17;9:84. doi: 10.1038/s42003-025-09349-1 (PMC12820229; doi:10.1038/s42003-025-09349-1)
Supplement: Supplementary file 4 — Reporting Summary [file 42003_2025_9349_MOESM4_ESM.pdf]

nature portfolio

Corresponding author(s): Sally J Dawson

Last updated by author(s): Nov 3, 2025

Reporting Summary

Nature Portfolio wishes to improve the reproducibility of the work that we publish. This form provides structure for consistency and transparency in reporting. For further information on Nature Portfolio policies, see our Editorial Policies and the Editorial Policy Checklist.

Statistics

For all statistical analyses, confirm that the following items are present in the figure legend, table legend, main text, or Methods section.

n/a	Confirmed	
☐	☒	The exact sample size (*n*) for each experimental group/condition, given as a discrete number and unit of measurement
☐	☒	A statement on whether measurements were taken from distinct samples or whether the same sample was measured repeatedly
☐	☒	The statistical test(s) used AND whether they are one- or two-sided<br>*Only common tests should be described solely by name; describe more complex techniques in the Methods section.*
☐	☒	A description of all covariates tested
☐	☒	A description of any assumptions or corrections, such as tests of normality and adjustment for multiple comparisons
☐	☒	A full description of the statistical parameters including central tendency (e.g. means) or other basic estimates (e.g. regression coefficient) AND variation (e.g. standard deviation) or associated estimates of uncertainty (e.g. confidence intervals)
☒	☐	For null hypothesis testing, the test statistic (e.g. *F*, *t*, *r*) with confidence intervals, effect sizes, degrees of freedom and *P* value noted<br>*Give P values as exact values whenever suitable.*
☒	☐	For Bayesian analysis, information on the choice of priors and Markov chain Monte Carlo settings
☒	☐	For hierarchical and complex designs, identification of the appropriate level for tests and full reporting of outcomes
☒	☐	Estimates of effect sizes (e.g. Cohen's *d*, Pearson's *r*), indicating how they were calculated

Our web collection on statistics for biologists contains articles on many of the points above.

Software and code

Policy information about availability of computer code

Data collection	All software used in data collection and analysis is standard commercial or opensource software and is described within the manuscript. This includes Tucker Davies Technology System 3 and BioSig RZ (v5.7.1) software for ABR collection, Matlab for ABR data analysis. Image acquisition and processing software : Fiji, Zen 3.0 SR software (Black edition), Airyscan, using Zen Blue software. Statistics were performed in GraphPad Prism.
Data analysis	All software used in data collection and analysis is standard commercial or opensource software and is described within the manuscript. This includes Tucker Davies Technology System 3 and BioSig RZ (v5.7.1) software for ABR collection, Matlab for ABR data analysis. Image acquisition and processing software : Fiji, Zen 3.0 SR software (Black edition), Airyscan, using Zen Blue software. Statistics were performed in GraphPad Prism.

For manuscripts utilizing custom algorithms or software that are central to the research but not yet described in published literature, software must be made available to editors and reviewers. We strongly encourage code deposition in a community repository (e.g. GitHub). See the Nature Portfolio guidelines for submitting code & software for further information.

Data

Policy information about availability of data

All manuscripts must include a data availability statement. This statement should provide the following information, where applicable:

- Accession codes, unique identifiers, or web links for publicly available datasets
- A description of any restrictions on data availability
- For clinical datasets or third party data, please ensure that the statement adheres to our policy

The datasets and materials used and/or analyzed during the current study are available on reasonable request to the authors , subject to an MTA where necessary. The Klhdc7bIMPC-/- mice are available from the IMPC. Regeneron materials described in this manuscript may be available to qualified, academic, non-commercial researchers upon request through our portal (https://regeneron.envisionpharma.com/ienv_research/visiontracker/portal/login.xhtml?pgm=ISR&windowId=4d6). Regeneron does not share clinical molecules. Regeneron does share alternative molecules that behave in a similar manner. For any questions about how Regeneron shares materials please connect with Regeneron using the preclinical collaborations email address (preclinical.collaborations@regeneron.com).

Research involving human participants, their data, or biological material

Policy information about studies with human participants or human data. See also policy information about sex, gender (identity/presentation), and sexual orientation and race, ethnicity and racism.

Reporting on sex and gender	n/a
Reporting on race, ethnicity, or other socially relevant groupings	n/a
Population characteristics	n/a
Recruitment	n/a
Ethics oversight	n/a

Note that full information on the approval of the study protocol must also be provided in the manuscript.

Field-specific reporting

Please select the one below that is the best fit for your research. If you are not sure, read the appropriate sections before making your selection.

☒ Life sciences ☐ Behavioural & social sciences ☐ Ecological, evolutionary & environmental sciences

For a reference copy of the document with all sections, see nature.com/documents/nr-reporting-summary-flat.pdf

Life sciences study design

All studies must disclose on these points even when the disclosure is negative.

Sample size	Sample sizes for comparisons were based on previous data (own experience and published data of others) used to detect significant differences in ABR data between mice with a hearing loss based and normal hearing mice of the same strain and age, Typically this would be between 5-9 mice in each group.
Data exclusions	None
Replication	The manuscript provides replication of findings in two independent Klhdc7b knockout models. Although there are small differences in some phenotypes between the 2 lines (which are discussed in the manuscript) they generally replicate the main findings for both.
Randomization	Samples were allocated by genotype. Both males and females were used and where possible equal numbers of males/females were chosen from each litter by genoytpe.
Blinding	Blinding was used where possible. For example in analysis of SEM samples and explant cultures, this was blinded. However, because samples were allocated by genotype it was not always possible to blind the experiment. It would also be apparent even a blinded experiment which mice had a severe hearing loss or which had greater hair cell loss and infer genotype.

Reporting for specific materials, systems and methods

We require information from authors about some types of materials, experimental systems and methods used in many studies. Here, indicate whether each material, system or method listed is relevant to your study. If you are not sure if a list item applies to your research, read the appropriate section before selecting a response.

Materials & experimental systems

n/a	Involved in the study
☐	☒ Antibodies
☒	☐ Eukaryotic cell lines
☒	☐ Palaeontology and archaeology
☐	☒ Animals and other organisms
☒	☐ Clinical data
☒	☐ Dual use research of concern
☒	☐ Plants

Methods

n/a	Involved in the study
☒	☐ ChIP-seq
☒	☐ Flow cytometry
☒	☐ MRI-based neuroimaging

Antibodies

Antibodies used	These are given in a Table in the supplementary methods - page 5-6.
Validation	The custom klhdc7b antibody was validated in the 2 knockout mouse models, data provided in Figure 2. Other antibodies are well established antibodies used in the inner ear as cell specific markers.

Animals and other research organisms

Policy information about studies involving animals; ARRIVE guidelines recommended for reporting animal research, and Sex and Gender in Research

Laboratory animals	Klhdc7bRegn KO mice were generated on the B6.CAST-Cdh23753A>G background, Klhdc7bIMPC-/-mice were generated on the C57BL/6N. This is clear in the manuscript.
Wild animals	n/a
Reporting on sex	Both sexes were used in the study. There was no difference in hearing loss between males and female mice in both knockout strains. Therefore, sex was not used as a basis for separate analysis in further functional and histological analysis.
Field-collected samples	n/a
Ethics oversight	All Regn mice were housed and treated according to guidelines from the Institutional Animal Care and Use Committee.<br>All IMPC mice were housed according to UK Home Office regulations. All procedures were licensed by the Home Office under the Animals (Scientific Procedures) Act 1986 and Amendment Regulations 2012 (Project Licence numbers PP7565374 and PP8324029) and approved by local institutional ethical review committees.

Note that full information on the approval of the study protocol must also be provided in the manuscript.

Plants

Seed stocks	n/a
Novel plant genotypes	n/a
Authentication	n/a
